# A New Paradigm for MAPK: Structural Interactions of hERK1 with Mitochondria in HeLa Cells

**DOI:** 10.1371/journal.pone.0007541

**Published:** 2009-10-22

**Authors:** Soledad Galli, Olaf Jahn, Reiner Hitt, Doerte Hesse, Lennart Opitz, Uwe Plessmann, Henning Urlaub, Juan Jose Poderoso, Elizabeth A. Jares-Erijman, Thomas M. Jovin

**Affiliations:** 1 Laboratory of Cellular Dynamics, Max Planck Institute for Biophysical Chemistry, Göttingen, Germany; 2 Departamento de Química Orgánica, Facultad de Ciencias Exactas y Naturales (FCEyN), Universidad de Buenos Aires (UBA), CIHIDECAR, CONICET, Buenos Aires, Argentina; 3 Proteomics Group, Max Planck Institute of Experimental Medicine, Göttingen, Germany; 4 Deutsche Forschungsgemeinschaft Research Center for Molecular Physiology of the Brain, Göttingen, Germany; 5 Transkriptomanalyselabor, University of Göttingen, Zentrum 3, Biochemistry and Molecular Cell Biology, Göttingen, Germany; 6 Bioanalytical Mass Spectrometry Group, Max Planck Institute for Biophysical Chemistry, Göttingen, Germany; 7 Laboratory of Oxygen Metabolism, University Hospital “Jose de San Martin”, UBA, Buenos Aires, Argentina; 8 Laboratorio Max Planck de Dinámica Celular, FCEyN, UBA, Buenos Aires, Argentina; Bauer Research Foundation, United States of America

## Abstract

Extracellular signal-regulated protein kinase 1 and 2 (ERK1/2) are members of the MAPK family and participate in the transduction of stimuli in cellular responses. Their long-term actions are accomplished by promoting the expression of specific genes whereas faster responses are achieved by direct phosphorylation of downstream effectors located throughout the cell. In this study we determined that hERK1 translocates to the mitochondria of HeLa cells upon a proliferative stimulus. In the mitochondrial environment, hERK1 physically associates with (i) at least 5 mitochondrial proteins with functions related to transport (i.e. VDAC1), signalling, and metabolism; (ii) histones H2A and H4; and (iii) other cytosolic proteins. This work indicates for the first time the presence of diverse ERK-complexes in mitochondria and thus provides a new perspective for assessing the functions of ERK1 in the regulation of cellular signalling and trafficking in HeLa cells.

## Introduction

ERK1 and ERK2 are members of the MAPK family of signalling proteins and play key roles in the transduction of extracellular stimuli into cellular responses [1], [2]. Induction of this signalling cascade leads to the phosphorylation of several target proteins that regulate cellular fate and other physiological processes [3]. The ultimate effects of ERK1/2 activation are determined by the phosphorylation of its downstream effectors located in the cytoplasm and nucleus, as well as in other cellular compartments. Indeed, the ubiquitous nature of ERK1/2 action is reflected in an ever-expanding list of ERK1/2 substrates [1]–[5]. The long-term actions of ERK1/2 are accomplished by promoting the expression of genes under the control of specific transcription factors, including Elk-1, Myc, Myb and the cAMP-response element. Activation of gene expression is preceded by the translocation of activated ERK1/2 from the cytoplasm to the nucleus [6], [7]. ERK1 and 2 are coexpressed in most tissues, are very similar in sequence and have been generally thought to be interchangeable. While recent evidence suggests that the ERK kinases are not functionally redundant and may have very different roles [see 8 for review], further studies are needed to assess the interplay between the two proteins and its effects on signalling dynamics. Increasing evidence suggests the existence of non-genomic effects of ERK. Shaul and Seger [4] showed that an alternative splice variant of ERK1 can participate in Golgi fragmentation during mitosis, while Klemke *et al.* [9] demonstrated that ERK phosphorylates myosin light chain kinase, a critical step in the regulation of myosin light chain function in contractility and cell migration. Poderoso *et al*. [5] observed that ERK1/2 activation by cAMP results in maximal steroidogenic rate.

ERK is present in mitochondria. Alonso *et al.* [10] reported the existence of ERK1/2 in the outer membrane and intermembrane space of brain mitochondria. The translocation of ERK1/2 to brain mitochondria follows a developmental pattern, peaking at stages E19-P2 and decreasing from P3 to adulthood. Baines *et al.* [11] have shown the presence of ERK in murine heart mitochondria and a PKCε-ERK module appears to play a role in PKCε-mediated cardioprotection. Poderoso *et al.* [5] reported the presence of ERK1/2 in the mitochondria of Leydig-transformed MA-10 cell line, and concluded that mitochondrial ERK activation was obligatory for PKA mediated steroidogenesis. Finally, Galli *et al.* [12] observed that ERK as well as p38, JNK and their respective MAPKKs are present in the mitochondria of a tumoral cell line, and furthermore that the traffic of these MAPKs in and out of the organelle is regulated by hydrogen peroxide. Although definite functional consequences of ERK localization in mitochondria have not been reported previously, one must consider that phospho-ERK1/2 may regulate mitochondrial activities related to cellular survival and metabolism. In the complex molecular infrastructure that underlies the mechanisms of activation and transduction, ERK orchestrates a series of signalling events that result in the recruitment of many downstream factors, including kinases, transcription factors, and other proteins. ERK present in mitochondria would be expected to form protein complexes involved in regulation of mitochondrial metabolism. Thus, identification of interaction partners of ERK in the organelle may provide important insights into the molecular basis of its putative role as a mitochondrial regulator.

Protein dimerization is common to numerous mechanisms for the transduction of extracellular signals. Phosphorylation of ERK2 facilitates its dimerization *in vitro*. By equilibrium sedimentation, Khokhlatchev *et al.* [13] determined that phosphorylated ERK2 sediments primarily as an 84 kDa species, whereas unphosphorylated ERK2 migrates as a mixture of a lesser fraction of 84 kDa and a predominant fraction of a 42 kDa species. A model of dimerized kinase deduced from the crystal structure of phosphorylated ERK2, proposes the physical basis for dimerization [14]. Phosphorylation leads to conformational changes in the two flexible regions of the protein molecule, the activation loop and the C-terminal extension, thereby generating the molecular interfaces for homodimerization. The two ERK2 molecules bind via a hydrophobic zipper complemented by two ion pairs, one on each side of the zipper [for reviews], [see 15 and 16]. ERK2 also dimerizes when transfected or microinjected into cells, but the process requires its phosphorylation [13]. Whether dimerization is necessary for nuclear localization remains controversial; some authors have observed an enhanced nuclear translocation upon phosphorylation of the kinase [13]. However, Lidke *et al.* (unpublished data of our laboratory) have determined that ERK mutants unable to dimerize retain the ability to translocate to the nucleus, albeit at a slower rate. Casar *et al*. [17] have recently demonstrated that scaffold proteins and ERK dimers are essential for the activation of cytoplasmic but not of nuclear substrates. Dimerization is critical for connecting the scaffolded ERK complex to cognate cytoplasmic substrates. In contrast, nuclear substrates associate with ERK monomers. Philipova and Whitaker [18] showed that ERK1 is capable of dimerizing both *in vivo* and *in vitro*. Dimerization *in vitro* of the human recombinant protein requires both ERK1 phosphorylation and the intervention of cellular cofactors. Exposure of the resultant high molecular weight complex to β-mercaptoethanol (β-ME) leads to dissociation. In view of the ambiguous structural and cell biological evidence for the existence and significance of dimerization, further investigation of the role of dimerization in the regulation of ERK trafficking under different cellular conditions is required.

The genome of animal mitochondria (mtDNA) encodes a small number of proteins, all of which are required for electron transport and oxidative phosphorylation, and the RNAs required for the translation machinery, i.e., two rRNAs and 22 tRNAs. The majority of the organelle proteins, including a large number of subunits of the electron transport complexes and the totality of the ribosomal proteins, are specified by nuclear genes [19]. Thus, the contributions of both the nuclear and mitochondrial genomes are required for maintaining functional oxidative phosphorylation, an appropriate balance of ribosomes and mRNAs, as well as intrinsic mitochondrial functions such as the synthesis of heme, purines and Fe-S clusters. From this perspective, the regulation of mtDNA transcription is an issue of major importance.

In the present study, we adopted a functional proteomic approach to gain insights into ERK-dependent signalling in mitochondria of HeLa cells. While both ERK1 and 2 are found in mitochondria, we focused our attention in this initial study on ERK1. We determined that ERK1 forms signalling complexes in the organelle and identified the components of these complexes. These proteins participate in multiple physiological pathways such as lipid metabolism, oxidative balance and anion transport. In addition, ERK interacts with histones and these nuclear proteins are present in the OMM of HeLa cells. We also evaluated the nature of ERK dimerization, and the role of the mitochondrion in this process. Finally, we assessed the potential role of ERK in the modulation of HeLa mitochondrial gene expression.

## Results

### Purity and integrity of the mitochondrial fraction

The procedures for the isolation of mitochondria from HeLa cells and the evaluation of purity are provided in the supporting online material (Fig. S1, Table S1 and Supporting Information S1). The mitochondrial preparations were intact according to standard criteria for assessing structural, morphological and functional integrity.

### Presence and translocation of hERK1 into cellular and isolated mitochondria

**Figure 1:**
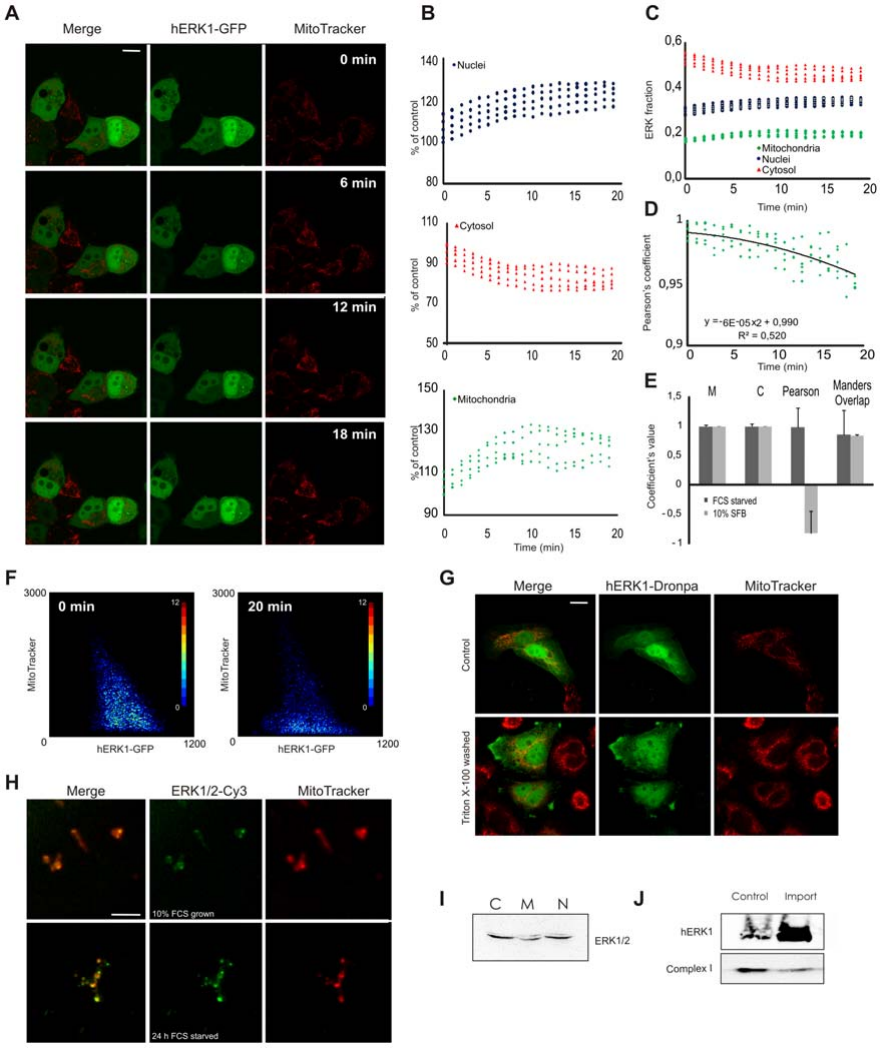
Presence and translocation of hERK1 into mitochondria. (A) HeLa cells transfected with hERK1-GFP, 24 h FCS starved and stained with MitoTracker CMXRos were stimulated with 10% FCS and fluorescence intensity of both green (GFP) and red (MitoTracker) channels was followed for 20 min in an Olympus FV1000 confocal microscope. Images of four representative time points after FCS stimulation of the individual and merged channels are shown. Bar = 10 µm. (B) Changes in hERK1-GFP fluorescence intensity after FCS stimulation analyzed in mitochondria, nuclei and cytosol for each of the 8 confocal planes analysed for the pair of images in A. Graphs show the net change displayed as percentage of the initial value (% of control) in each compartment after FCS stimulation. (C) Redistribution of hERK1-GFP fluorescence intensity in the different cellular compartments upon FCS stimulation. (D) Change in Pearson's correlation coefficient after FCS stimulation of serum starved cells analyzed within the mitochondrial mask. (E) Colocalization coefficients for HeLa cells FCS starved or cultured in 10% FCS. The. M and C coefficients were calculated only for red images. Manders Overlap = Manders Overlap coefficient. (F) hERK1-GFP and MitoTracker intensity correlation plots 0 and 20 min after FCS stimulation. Color bar = number of pixels. (G) HeLa cells transfected with hERK1-Dronpa, stained with MitoTracker Deep Red and washed in 0.1% Triton X-100 buffer (lower panel). An image of the individual and merged channels is shown compared to non-Triton washed cells (upper panel). (H) Isolated mitochondria from serum starved HeLa cells (lower panel) or cells cultured in 10% FCS (upper panel). Cells were labelled with MitoTracker Deep Red and further fixed and immuno-stained for phospho-ERK. Secondary antibody was conjugated to Cy3. An image of the individual and merged channels is shown for each case. (I) Western blot of different HeLa cellular fractions probed against ERK1/2. C = cytosol, M = mitochondria, N = nuclei. (J) Mitochondria from HeLa incubated with hERK1 recombinant protein in an import assay, and analyzed by western blotting. Loading control, antibody to a mitochondrial complex 1 subunit.

In this work we demonstrated the presence of endogenous and recombinant hERK1 in purified non-contaminated mitochondria of HeLa cells. HeLa cells were transfected with hERK1-GFP and further stained with a specific mitochondrial marker, MitoTracker RED CMXRos, and analyzed by confocal microscopy. Approximately 15% of the hERK1-GFP molecules localized in mitochondria, 30% in the nuclear compartment and the rest were distributed in cytosol in 24 h serum starved HeLa cells (Fig. 1, A–C). Mitochondrial hERK1-GFP content was greatly reduced in the organelles of not serum deprived cells (Fig. S2). Stimulation with fetal calf serum (FCS) led to a redistribution of hERK1-GFP among the different subcellular compartments (Fig. 1, A–F and Movie S1). The predominant effect was a net import of ERK into the nucleus with the corresponding depletion of the cytosolic pool. An additional early event was the entry of ERK into mitochondria, followed by an exit synchronized with the translocation to the nucleus (Fig. 1, A–F and Movie S1). The interdependence of nuclear and mitochondrial ERK was revealed by analysis of the slopes of the redistribution curves during 7–20 min (Fig. 1B). The rate of ERK entry into the nuclear compartment (0.5% of initial value/min) was approximately equal to the sum of ERK depletion from the mitochondria and cytosol (−0.5 and −0.1%/min, respectively). No hERK1 redistribution was observed in the absence of FCS stimulation (Fig. S3, and Movie S2). We did not observe a similar redistribution of GFP protein in HeLa cells under the same conditions (Fig. S4). Several reports show that after cellular starvation ERK1/2 are responsive to proliferative stimuli [12], [20], including the addition of FCS (Lidke et al., unpublished data of our laboratory). However, response to these stimuli is abrogated in cells under normal culture conditions favoring proliferation (data not shown).

We evaluated the colocalization indexes of the hERK1-GFP and MitoTracker images within the mitochondrial mask either in 24 h FCS deprived HeLa, or in cells grown in complete media (10% FCS); and the change in Pearson's correlation coefficient upon FCS stimulation of 24 h starved cells (Fig. 1, D–E; for details of the analysis see supplemental online material and references therein). The coefficients M and C were unity, indicating that the totality of the pixels enclosed by the mitochondrial mask contained hERK1-GFP (Fig. 1E). The Pearson's correlation coefficient decreased after FCS stimulation as ERK moved out of the organelle (compare Fig. 1B, lower panel and D). The diminished correlation between hERK1-GFP and MitoTracker fluorescence upon FCS stimulation was also observed in the intensity correlation plots in figure 1F. At time 0, most of the pixels displayed both green and red fluorescence intensities; upon stimulation the diagonal was lost and pixels were located along the hERK1-GFP axis with only a few pixels with high red fluorescence intensity and variable GFP intensity. We evaluated the significance of the Pearson's correlation coefficient of hERK1-GFP and MitoTracker RED CMXRos images according to previously reported procedures [21]. The value was significant, arguing for true colocalization instead of a fortuitous superposition of randomly distributed fluorophores (Fig. S5 and Supporting Information S1). The Pearson's correlation coefficient for HeLa cells grown continuously in 10% FCS was negative (Fig. 1E), indicating a lack of colocalization.

To demonstrate unequivocally the presence of hERK1 in HeLa mitochondria, we transfected cells with hERK1-Dronpa and labelled with MitoTracker Deep Red. The cells were washed with 0.1% Triton X-100 to eliminate cytosolic ERK. We observed hERK1-Dronpa in the mitochondria even after the Triton wash (Fig. 1G).

Endogenous ERK1/2 was also present in the mitochondria of HeLa cells (Fig. 1, H–I). We incubated isolated mitochondria with MitoTracker Deep Red and fixed and labelled them with an antibody against phospho-ERK1/2 and a second antibody conjugated with Cy3. Confocal microscopy revealed that phosphorylated ERK1/2 was present in the mitochondria (Fig. 1H). Indeed, we determined a 3-fold increase in the signal of phospho-ERK1/2 in 24 h serum deprived cells prior to mitochondria isolation compared to non-serum starved cells (fluorescence intensity in arbitrary units: 430±120 vs. 90±45, n = 15 and n = 32, respectively, p<0.05, Student's *t* test). The presence of endogenous ERK1/2 in the mitochondria was corroborated by western blotting (Fig. 1I; see Fig. 1S for mitochondrial purity and fractionation markers). In addition, incubation of mitochondria with the recombinant protein hERK1 in an import assay led to entry of the protein into the organelle (Fig. 1J).

The above findings are consistent with the report of Galli *et al.* [12] of a redistribution of ERK1/2 and recombinant ERK2 between the subcellular compartments of a lung cancer cell line, LP07, upon stimulation with H_2_O_2_. Altogether, these data strongly suggest that upon proliferative stimulation, ERK1 translocation to the nucleus ensues from both the mitochondrial and cytosolic pools of the kinase.

### hERK1 interaction with the mitochondrial proteome

**Figure 2:**
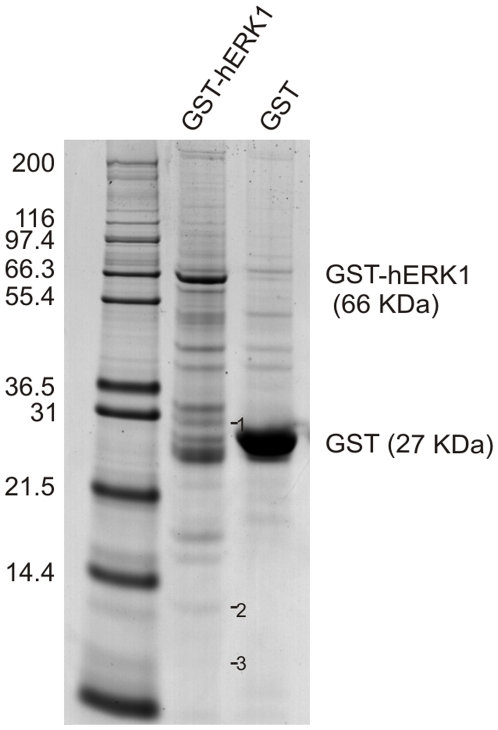
hERK1-GST pulldown assay. GST-hERK1 or GST-null recombinant proteins immobilized on GSH-agarose were incubated overnight with a mitochondrial extract. Beads were extensively washed and eluted proteins were run on SDS-PAGE. Proteins were stained with Coomasie Brilliant Blue G-250. Molecular weight markers displayed on the left. GST-hERK1 and GST-null recombinant proteins highlighted on the right. Numbers on the right side of the bands recovered in GST-hERK lane represent proteins identified by mass spectrometry and shown in Table 1.

The presence of ERK in mitochondria and the response to serum growth factors argue for a putative regulatory role of ERK1/2 in mitochondrial metabolism and we addressed this issue by means of interaction assays of GST-hERK1 recombinant protein on pure mitochondrial preparations, followed by proteomic analysis. Mitochondrial extracts were incubated with GST-hERK1 or GST-null recombinant proteins and extensively washed. The complexes were resolved by SDS-PAGE under denaturing conditions and Coomasie stained (Fig. 2). Most of the bands appeared in the GST-hERK1 lane, attesting to the specificity of the interaction. In every case, a band in the GST-null lane was taken as a control. Only proteins that appeared in the GST-hERK1 but not in the GST-null lanes were considered to be specific ERK interactors. We detected the formation of complexes with several mitochondrial proteins, described in Table 1, Table S2, in the following sections and in the supplemental online material.

**Table 1:** hERK1 signaling complexes in mitochondria from HeLa cells.

	MW (kDa)	Known function	Accesion No	Cellular Localization
***Transport proteins***				
Porin 31HL (VDAC1)	30.7^(1)^	Transport of anions, cations, ATP, Ca^2+^ and other metabolites between mitochondria and cytosol. Potential role in protein export from mitochondria.	gi|238427	OMM, plasma membrane, caveolae and caveolae like domains [23]
***Structural proteins***				
Histone H2A*	14.2^(2)^	Nucleosome assembly	gi|24638446	Nuclei
Histone H4*	11.3^(3)^	Nucleosome assembly	gi|51315727	Nuclei

GST-hERK1 or GST-null recombinant proteins immobilized on GSH-agarose were incubated overnight with mitochondrial extracts. Beads were extensively washed and eluted proteins run on PAGE-SDS. Proteins were stained with Coomasie Brilliant Blue G-250 (Fig. 2). A sample of each band in the GST-hERK1 or GST-null lane was excised and analyzed by mass spectrometry for protein identification. Only proteins present in GST-hERK but not in GST-null precipitates were considered as specific ERK partners. *Also confirmed by LC-MS/MS. ^(#)^Numbers in superscript correspond to the numbering of protein bands in Fig. 2. Only ERK interaction partners that were extensively corroborated by further experiments are shown. For the totality of ERK interaction partners recovered after GST-hERK1 pulldown and MALDI-TOF identification refer to supplemental Table S2 and Supporting Information S1.

### Transport proteins

Porin 31HL or VDAC1 (voltage-dependent anion channel 1) is a 31 kDa protein comprised of one polypeptide chain with hydrophobic and hydrophilic domains [22]. It is the major transport protein of the OMM [23]. Monomeric VDAC serves itself as a functional channel [24]. However, there is evidence that it can form dimers and possibly tetramers [25], [26], and that this oligomerization may be involved in mitochondrial mediated apoptosis [25], [27]. The OMM provides a barrier between the mitochondrial inner membrane and cytoplasm and VDAC mediates the complex interactions between mitochondria and other parts of the cell by transporting anions, cations, ATP, Ca^2+^ and metabolites. Thus, VDAC plays an important role in the coordination of communication between mitochondria and cytosol, in which the formation of transient complexes of VDAC with other proteins is an essential feature. Although the mechanism remains elusive, several hypotheses have been proposed [26]–[30].

**Figure 3:**
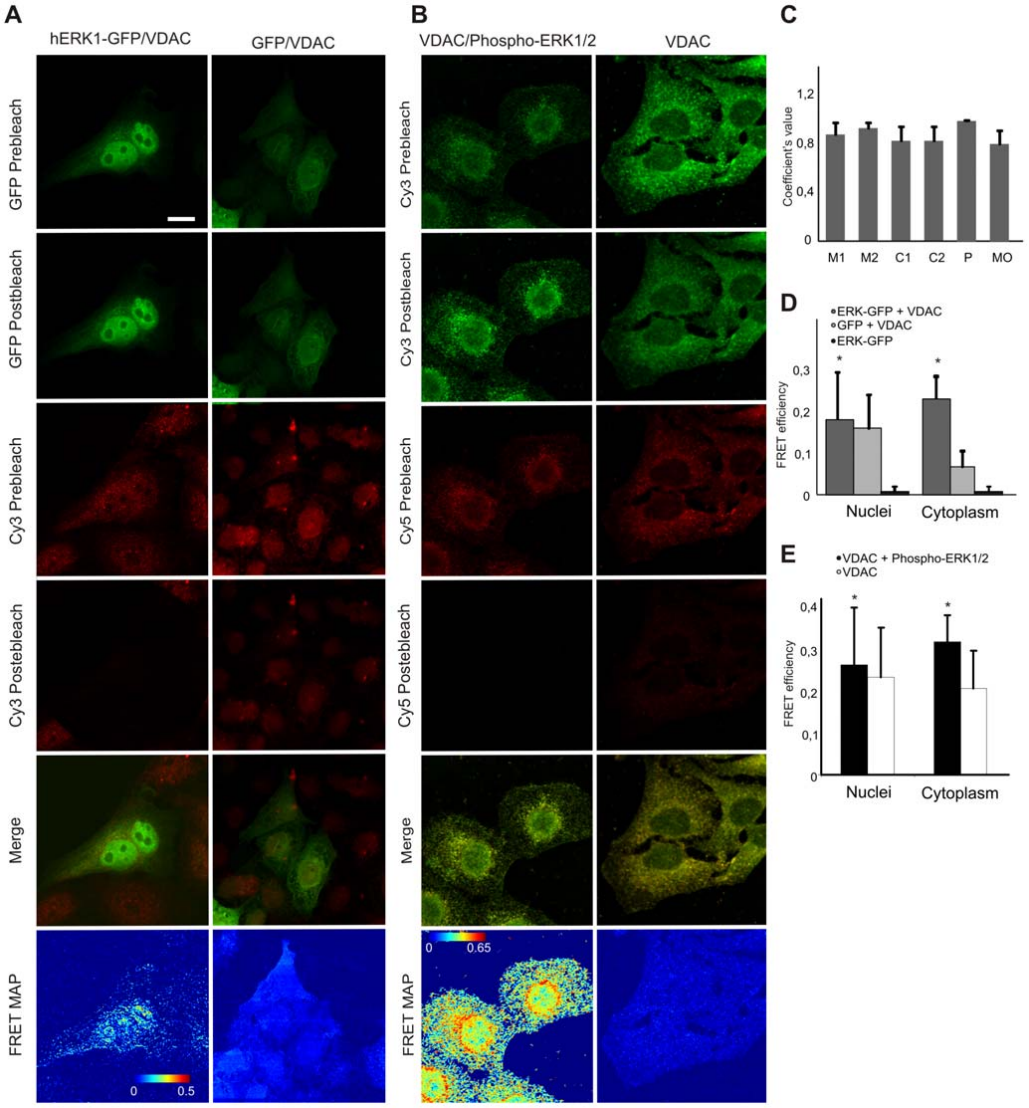
ERK interaction with VDAC. (A) HeLa cells transfected with hERK1-GFP, fixed and labelled against VDAC. Secondary antibody was conjugated with Cy3. The GFP and Cy3 images prior and after Cy3 photobleaching, the merged image of GFP and Cy3 prior to photobleaching and a FRET map according to Eq. 1 are shown. Bar = 10 µm. Color bar = FRET efficiency. Negative control: the same FRET experiment in HeLa cells transfected with GFP and further stained against VDAC as above. Representative images shown on the right. (B) HeLa cells fixed and stained with antibodies to phospho-ERK and VDAC. Secondary antibodies conjugated to Cy5 and Cy3, respectively. Cy3 and Cy5 images prior and after Cy5 photobleaching, a merged image of Cy3 and Cy5 prior to photobleaching and a FRET map in accord to Eq. 1 are shown. Bar = 10 µm. Color bar = FRET efficiency. Negative control: HeLa cells labelled against VDAC with both secondary antibodies; image series shown on the right. (C) Colocalization indexes estimated for HeLa cells transfected with hERK1-GFP and labelled for VDAC as in (A). M and C coefficients were estimated for both green and red channel images (M_green_, M_red_, C_green_, C_red_). P = Pearson's correlation coefficient, MO = Manders Overlap Coefficient. (D) Mean FRET efficiency in HeLa cells transfected with hERK1-GFP and labelled for VDAC as in (A) in accord to Eq. 1. Negative controls: HeLa cells transfected with GFP and labelled against VDAC plus Cy3 for specificity (light grey bars) and HeLa cells transfected with hERK1-GFP and no further staining for a FRET negative value (black bars); *p<0.5 with respect to GFP transfected cells. (E) Mean FRET efficiency in HeLa cells labelled for phospho-ERK and VDAC plus Cy5 and Cy3, respectively, in accordance with Eq. 1. Negative controls: HeLa cells labelled with antibody to VDAC plus both secondary antibodies for specificity (white bars); *p<0.5 with respect to negative control (Student's *t* test).

Mass spectrometric protein identification revealed an interaction of hERK1 with VDAC1 in mitochondria (Fig. 2 and Table 1). We observed an extensive colocalization of hERK1-GFP with VDAC in HeLa cells (Fig. 3A). Greater than 80% of pixels in the cell contained fluorescence in both channels (C_green_ and C_red_), and Pearson's correlation coefficient was almost 1 (Fig. 3C). We corroborated hERK1 and VDAC1 interaction by confocal microscopy utilizing FRET (Förster Resonance Energy Transfer) between hERK1-GFP transfected into HeLa cells and Cy3-conjugated secondary antibody used to immuno-localize VDAC1 with a specific primary antibody. The mean FRET efficiency between these two molecules in the cytoplasm was 0.3 and the energy transfer was largely confined to the perinuclear zone (Fig. 3, A and D). The interaction also occurred in intact cells, a finding established by fixing and labelling cells with primary antibodies against phospho-ERK1/2 and VDAC1 and secondary antibodies conjugated to Cy5 and Cy3, respectively, and conducting comparative FRET experiments. Energy transfer was observed supporting the idea of an interaction between these molecules in cytoplasm and nuclei, but mainly in the perinuclear zone (Fig. 3, B and E). Little FRET was apparent in the negative control although some unspecific Cy5 signal was present.

The mitochondrial network is disposed primarily in the perinucelar region. Our findings support the notion that ERK1 may translocate to and from the organelle through the VDAC channel, a possibility that deserves further study as it could constitute a mechanism for the regulation of ERK availability to the nucleus, and thus its influence on cell fate.

VDAC1 Ser 104 is embedded in a minimal phosphorylation motif for ERK (Ser/Thr- Pro) [31], and thus may serve as a putative target for ERK1 phosphorylation. In addition, the study of VDAC amino acid sequence shows that the molecule has a potential ERK docking domain (Fig. S6) [32], [33]. If ERK1 can bind and phosphorylate VDAC, it could regulate channel activity by modulating the traffic of metabolites between mitochondria and cytosol.

### Structural proteins

**Figure 4:**
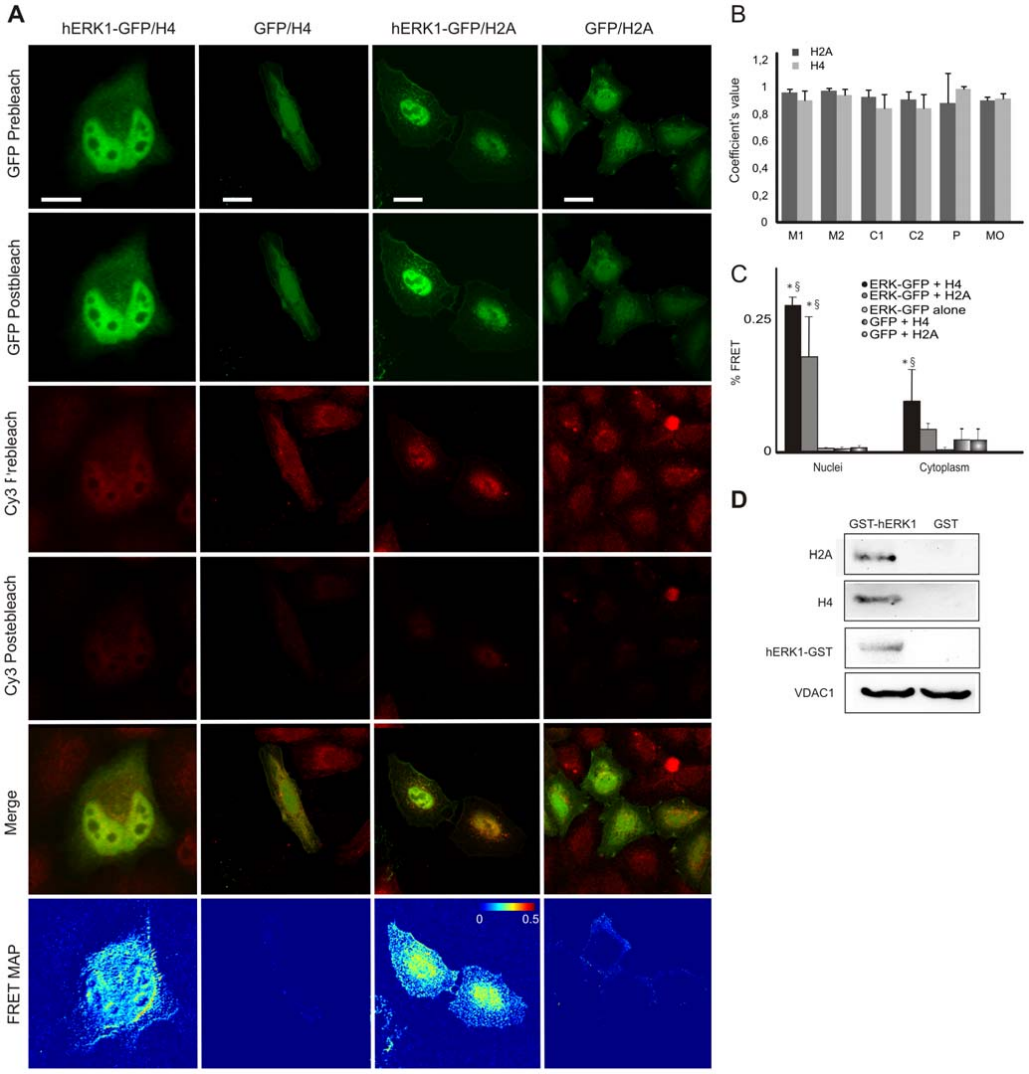
hERK1 interaction with histones. (A) HeLa cells transfected with hERK1-GFP, fixed and labelled with antibodies to H2A and H4 and a secondary antibody conjugated with Cy3. GFP and Cy3 images prior and after Cy3 photobleaching, a merged image of GFP and Cy3 prior to photobleaching and a FRET map in accord to Eq. 1 are shown. Bar = 10 µm. Color bar = FRET efficiency. Negative control: same FRET experiment in HeLa cells transfected with GFP and further stained against histones as above. Representative images are shown. (B) Colocalization indexes. M and C coefficients were estimated for both green and red images (M_green_, M_red_, C_green_, C_red_). P = Pearson's correlation coefficient, MO = Manders Overlap Coefficient. (C) Mean FRET efficiency estimated and analyzed as in Fig. 3; *p<0.5 with respect to GFP transfected cells. §*p*<0.5 with respect to hERK1-GFP transfected cells with no histone or Cy3 staining. (D) Pulldown experiment performed as in Fig. 2, with proteins detected by western blotting.

**Figure 5:**
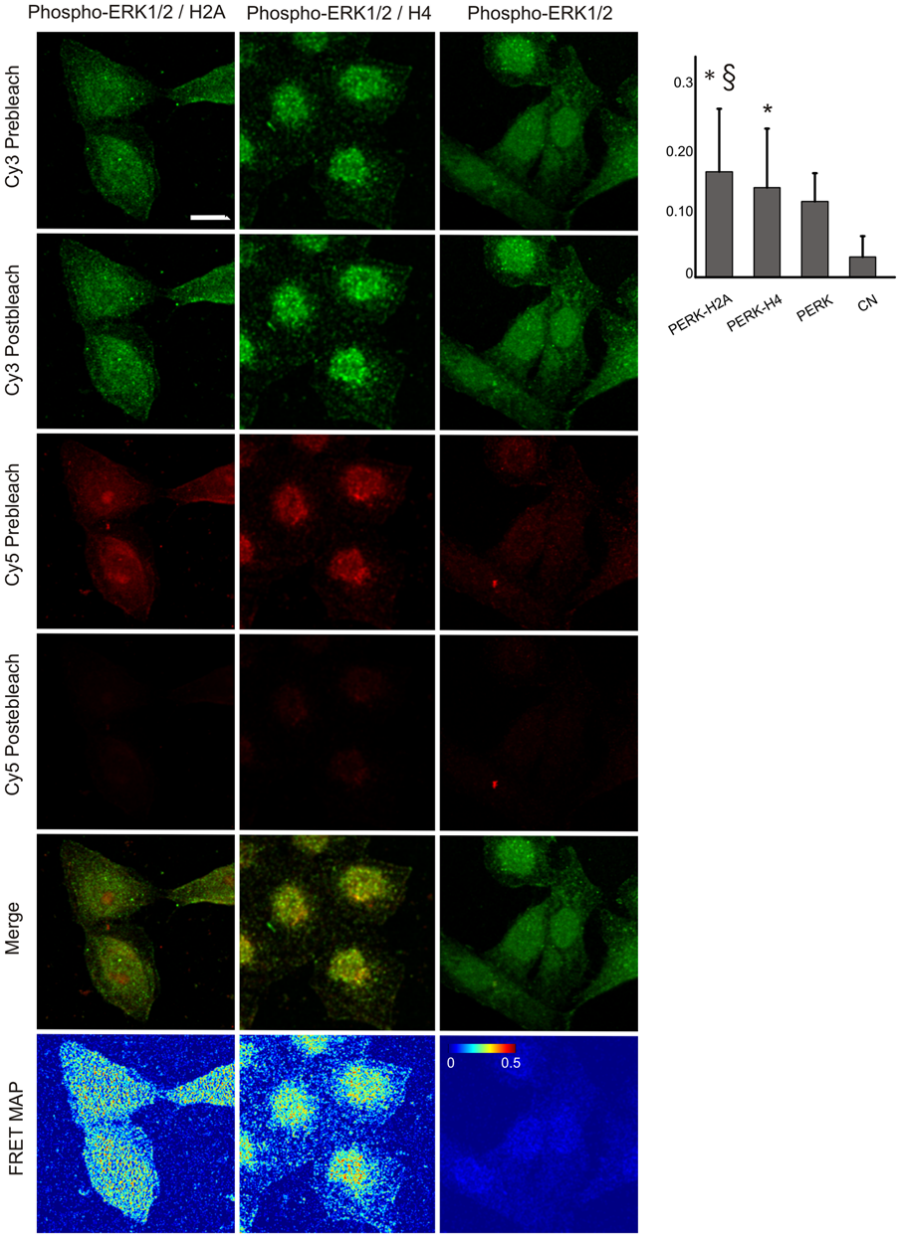
Endogenous ERK1/2 interaction with histones. HeLa cells fixed and stained against phospho-ERK and histones. Secondary antibodies conjugated to Cy3 and Cy5 respectively. Cy3 and Cy5 images prior and after Cy5 photobleaching, a merge image of Cy3 and Cy5 prior to photobleaching and a FRET map in accordance with Eq. 1 are shown. Bar = 10 µm. Color bar = FRET efficiency. Control for specificity of ERK-histones interaction: HeLa cells labelled with antibody to phospho-ERK and both secondary antibodies (PERK); representative images are shown. Negative FRET control: HeLa cells labelled for phospho-ERK and secondary Cy3 antibody (CN). Mean FRET efficiency shown in the bar graph on the upper right side; *p<0.5 respect to negative control and §*p*<0.5 with respect to specificity control (ANOVA and Scheffé test).

Using MALDI-TOF-MS, we identified histones H2A and H4 as binding partners of ERK in five out of five pulldown assays performed (Table 1 and Fig. 2). These results were confirmed by LC-MS/MS, a complementary approach for protein identification. We then determined whether these proteins were truly integrated into mitochondria, or merely attached to the OMM. By flow cytometry we observed the presence of H2A and H4 in the mitochondrial fraction, either with or without permeabilization of the mitochondrial membrane, a result compatible with a superficial distribution of histones on the organelle (Fig. S7, A). Negative controls were performed with mitochondria alone or stained with Cy5 in the absence of primary antibodies. Both yielded negative results, with a fluorescence intensity mean lower than 6. By western blotting we detected the presence of histones in the mitochondrial fraction, but these proteins were removed after treatment of mitochondria with proteinase K, further supporting a localization of the proteins in the OMM (Fig. S7, B). We observed extensive colocalization throughout the cell of hERK1-GFP and H2A or H4; 85–90% of the pixels inside the cell contained fluorescence intensity in both channels and Pearson's correlation coefficient was 0.99 for H4 and almost 0.9 for H2A (Fig. 4, A–B). We corroborated the interaction of hERK1 with H2A and H4 by pulldown of GST-hERK1 and immuno-staining of histones (Fig. 4D) and observed a FRET efficiency of almost 0.2 and 0.3 between hERK1-GFP and H4 or H2A, respectively, in the nuclei of HeLa cells, with a somewhat lower in the cytoplasm (Fig. 4C). To asses whether these biochemical interactions occurred in intact cells, we conducted comparative FRET experiments in HeLa labelled with antibodies to phospho-ERK1/2 and H2A or H4 and secondary antibodies conjugated to Cy3 and Cy5, respectively. The mean FRET efficiency was 0.17 for the ERK-H2A interaction and 0.14 for the ERK-H4 interaction. The former value was significantly greater than that of the specificity control (0.12, HeLa cells labelled against phospho-ERK1/2 plus both secondary antibodies), and both interactions were significantly greater than in the negative control (Fig. 5, HeLa cells labelled against phospho-ERK1/2 plus Cy3).

Both H2A and H4 have Ser and Thr in their sequence, but none of them is present in a canonical ERK phosphorylation motif. However, Ser 2 in H2A, and Ser 2 and Ser 48 in H4 are followed by a glycine, motif that has also been reported to be recognised and phosphorylated by ERK [33]. In addition, both H2A and H4 amino acid sequences display putative ERK docking domains (Fig. S6) [32], [33].

### ERK dimerization

**Figure 6:**
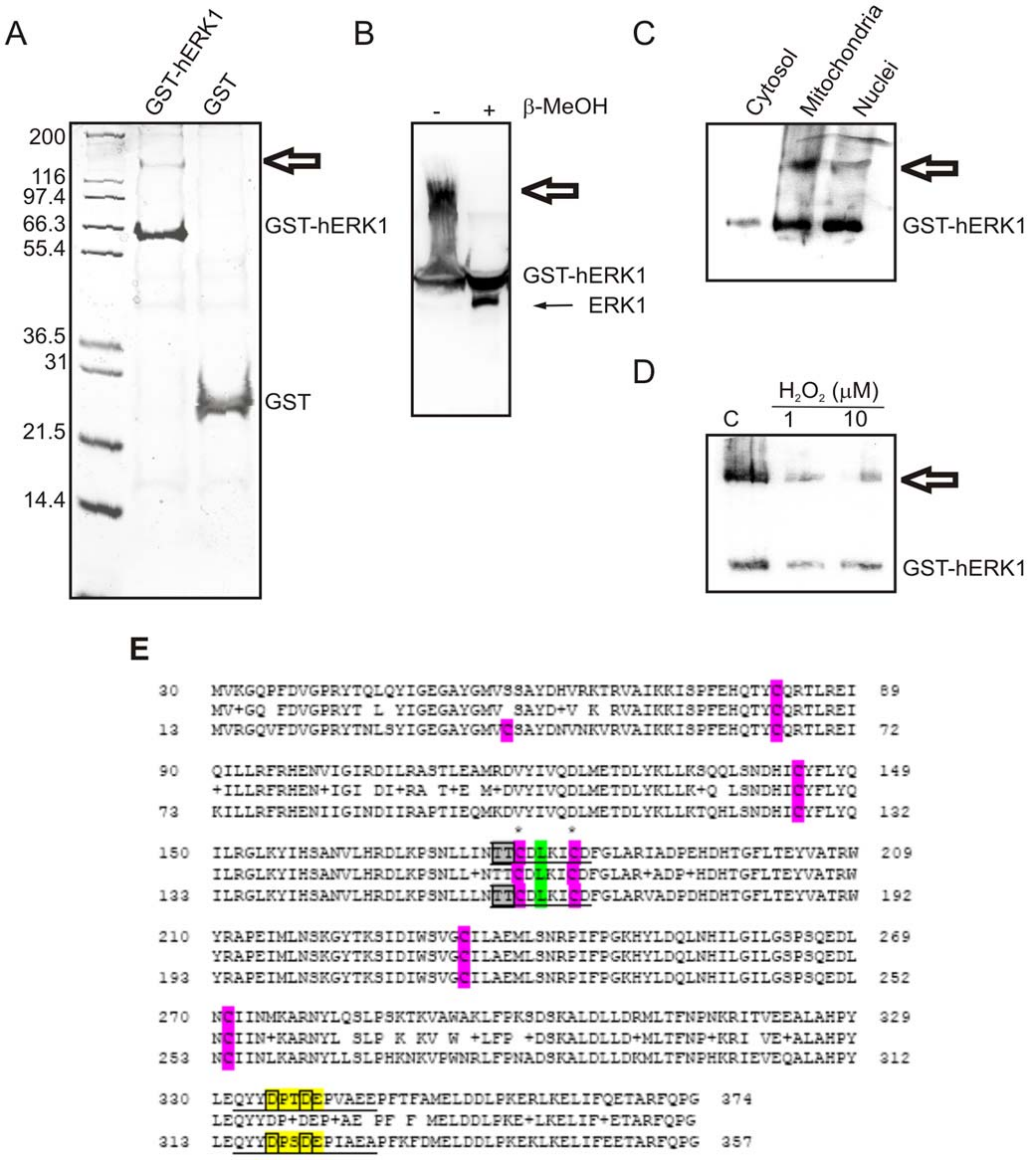
ERK dimer formation in mitochondria. (A) Pulldown assay and electrophoresis as in Fig. 2. Samples were run in the absence of β-ME or DTT. Molecular weight markers displayed on the right. Thick arrow, GST-hERK1 dimer. (B) Recombinant hERK1-GST immobilized on GSH-agarose incubated with mitochondria with dimerization evaluated by western blotting in the presence or absence of β−ME. Thin arrow, endogenous ERK, recovered from the dimer upon incubation with β−ME. (C) hERK1-GST recombinant protein immobilized on GSH-agarose and incubated with mitochondrial, cytosolic or nuclear fractions. hERK1-GST and hERK1-GST dimer detected by western blot. (D) GST-hERK1 recombinant protein oxidized with different H_2_O_2_ concentrations and processed as in (C). Dimerization evaluated by western blotting. (E) Alignment of ERK1 (top) and ERK2 (bottom) amino acid sequences. CD domain (yellow), ED domain (grey), dimerization site (green) [34], [35], cysteines (purple) and cysteines (*) putatively involved in disulfide bond formation are highlighted.

As stated earlier, ERK1 forms homodimers [13], [14], [19], and we confirmed the presence of dimers inside mitochondria. After pulldown experiments under high stringency conditions and electrophoresis in the absence of β-ME or DTT, a high molecular weight band (MW ∼130; Fig. 6A) was detected and identified as a dimer of GST-hERK1 by MALDI-TOF-MS. This band was disrupted after treatment with β-ME: in figure 6B, the high molecular weight band corresponding to hERK1-GST dimer (left lane) disappeared after β-ME treatment (right lane). This result suggests the presence of a disulfide bridge between the two monomers. The latter would involve ERK cysteines rather than GST residues since we recovered endogenous ERK after treatment of GST-hERK1 dimers with β-ME (Fig. 6B, thin arrow), indicating that endogenous ERK is also able to form a covalent dimer.

We investigated whether all the cellular fractions were capable of enabling ERK dimerization by conducting comparative pulldown experiments with mitochondrial, nuclear and cytosolic extracts. GST-hERK1 dimerization was favoured in the mitochondria, also occurred in the nuclei, but was hardly detectable in the cytosol (Fig. 6C). Inasmuch as the redox state of the cell regulates kinase activity [12], we determined whether oxidation could alter the dimerization capacity of hERK1. Oxidation decreased the formation of the dimer (Fig. 6D), a result attributable to the oxidation of cysteine residues to sulfonic acid or other derivatives, reactions that would impede disulfide bridge formation.

The cysteine residues involved in the bridge formation in hERK1 were identified by MALDI-ToF/ToF and ESI-LC-MSMS analysis of the band corresponding to the dimer either in the presence or absence of DTT. The off-line MALDI-ToF/ToF and ESI-LC-MSMS experiments from the DTT treated samples reproducibly revealed two peptides, positions 72 to 84 (KISPFEHQTY**C**QR) and 166 to 189 (DLKPSNLLINTT**C**DLKI**C**DFGLAR) in ERK1 that contain cysteines (Cys 82, 178 and 183). These two peptides were not observed and thus were not sequenced in the non-DTT treated sample. The results suggest that these protein regions may be involved in forming disulfide bridges. We developed 6 mutants of hERK1, changing each of the six cysteines present in the molecule by alanine. All mutants were capable of dimerization (data not shown), suggesting the participation of more than one cysteine in disulfide bridge formation. It is noteworthy that both Cys 178 and 183 are embedded in the ERK ED domain involved in protein-protein interactions between ERK and its kinases and substrates (Fig. 6E) [34], [35]. This domain also participates in homodimerization. Further studies are needed to delineate the physiological relevance of ERK dimerization occurring in mitochondria.

### Regulation of mitochondrial gene expression

**Figure 7:**
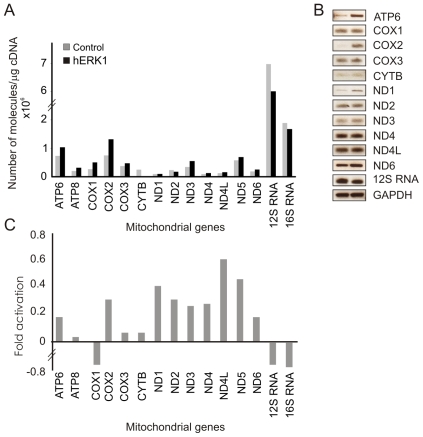
ERK modulatory effect on mitochondrial gene expression. Mitochondria from HeLa cells isolated and incubated with hERK1 for 1 h at 37°C in an *in organello* RNA synthesis assay (see experimental procedures). mtRNA isolated and every mitochondrial gene expression evaluated by (A) Real time PCR, or (B) Semiquantitative PCR. GAPDH used as housekeeping gene. (C) Mitochondrial gene expression also studied by microarray (see experimental procedures). Mean fold-activation for every gene after ERK treatment of mitochondria (See also Table 2). ND: NADH dehydrogenase; ATP: ATP synthase; COX: cytochrome oxygenase. CYTB: cytochrome *b*. Number indicates subunit.

The ability of hERK1 to modulate mitochondrial transcription was analyzed by microarrays, and both Real Time and semiquantitative PCR experiments. Mitochondria from HeLa cells were isolated and incubated with hERK1 for 1 h at 37°C in an import assay established for *in organello* RNA synthesis [36] (see experimental procedures). mtRNA was further isolated and every mitochondrial gene expression was evaluated as previously mentioned. Almost every mitochondrial gene exhibited a tendency for up regulation by treatment with hERK1. In contrast, the mitochondrial rRNA genes showed a tendency for down regulation. The available evidence raises the distinct possibility that ERK exerts a modulatory role on mitochondrial gene transcription (Fig. 7 and Table 2). The amino acid sequence of mitochondrial transcription factor A (TFAM), contains a potential ERK docking domain (Fig. S6) [32], [33] and Ser 31, Ser 177 and Thr 122 immersed in an ERK phosphorylation motif, suggesting that ERK could bind and activate the transcription factor and thereby regulate mitochondrial gene expression.

**Table 2:** Mitochondrial gene expression studied by microarray.

Mitochondrial gene	Fold activation	Significance	Mean fold activation Mean±SD
ND1	0.5; 0.6; 0.7	0.0007; 0.0009; 0.04	0.4±0.3
ND2	0.6	0.0001	0.3±0.3
ND3	0.5	0.08	0.3±0.2
ND4	0.6	0.03	0.3±0.2
ND4L	0.6; 0.8; 1.5	0.002; 0.02; 0.006	0.6±0.5
ND5	0.7; 0.7; 0.8	0.000; 0.006; 0.04	0.5±0.4
ND6	-	1	0.2±0.3
ATP6	-	1	0.3±0.1
ATP8	-	1	0.03±0.3
COX1	−1.3; −1; −0.6	0.01; 0.0003; 0.002	−0.7±0.4
COX2	0.5	0.001	0.3±0.2
COX3	-	1	0.07±0.2
Cytochrome c	-0.6; 0.9	0.1; 0.005	0.07±0.5
12S RNA	−0.9	0.05	−0.2±0.4
16S RNA	-	1	−0.2±0.2

The microarray contained 6 different oligos per mitochondrial gene as described in Materials and Methods. The activation fold for each oligo was determined and reported as Mean±SD. The fold-activation value is given only in cases with statistical significance, reported as *p*, tested as described in Materials and Methods and deemed positive for values ≤0.05. Positive values correspond to an up regulation upon hERK1 treatment. ND: NADH dehydrogenase; ATP: ATP synthase; COX: cytochrome oxygenase. Number denotes subunit.

## Discussion

To our knowledge, this is the first investigation exploiting a functional proteomic strategy for systematically delineating hERK1 signalling complexes in mitochondria. Using this approach we have demonstrated the physical associations of hERK1 with structural, signalling, transport and metabolic proteins in the mitochondria of HeLa cells. Most of the proteins found to be physically associated with ERK were not previously known as interaction partners of this kinase.

**Figure 8:**
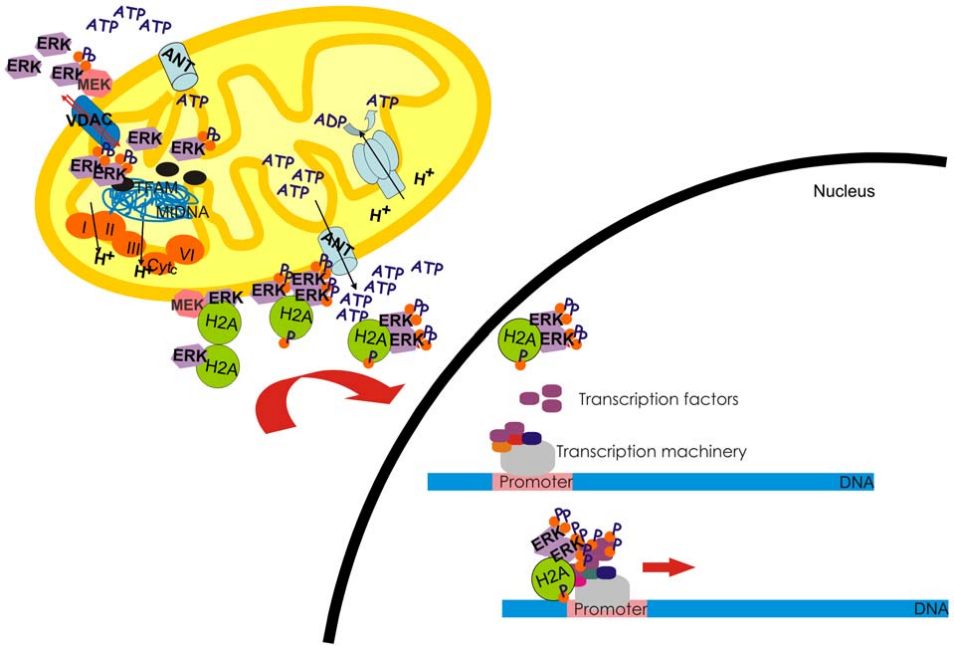
Postulated structural interactions of hERK1 with mitochondria. Scheme showing a new perspective for the physiological role of hERK1 in relation to the regulation of cellular signalling and trafficking in HeLa cells. ERK1 may be a carriage protein that would facilitate histone traffic to the ATP source in the surroundings of the OMM, and afterwards to the nuclei. Altogether this complex may interact with ERK transcription factors and the transcription machinery to enhance RNA synthesis. ERK translocation into and out the mitochondria may occur via VDAC protein. Once inside, the kinase may regulate mtDNA transcription by regulation of TFAM. I, II, III and IV: mitochondrial electron transport chain complexes, Cyt: cytochrome *c*, ANT: ATP carrier. The model is simplified for H2A, but the same is proposed for H4.

We found that ERK interacts with histones, and confirmed the presence of these nuclear proteins in the proximity of the OMM in HeLa cells (Fig. S7). We performed a variety of contamination controls to establish the integrity and purity of the mitochondrial fraction (Fig. S1, Table S1 and Supporting Information S1). Nonetheless, further research is required to ascertain the physiological relevance of ERK-histone complexes in, on and near mitochondria. Perhaps the best-characterized direct link between signal transduction and chromatin modification is seen in mammalian cells upon mitogen stimulation. H3 and other chromatin-associated proteins, such as HMG14, are rapidly and transiently phosphorylated in response to stimulation by epidermal growth factor (EGF) [37]. Phosphorylation occurs at serine 10 of the H3 N-terminal tail, and the time course of H3 phosphorylation closely corresponds to the transient expression of activated immediate-early genes, suggesting that this histone modification is linked to transcriptional activation [reviewed in 37]. In the same sense, phosphorylation of a yeast histone H2A at the C-terminal serine 129 is of central importance in double-strand break repair. Phosphorylation destabilizes chromatin structures and thereby facilitates access of repair proteins [38]. Our findings support the notion that histones H2A and H4 are bound to hERK1 in mitochondria of HeLa. In the single previous study of histones in mitochondria, Okamura et al. [39] reported that H1.2 is located in mitochondria of SCCTF cells along with BAK. Furthermore, Konishi et al. [40] suggested that H1.2 translocation from nuclei to cytosol transduced the apoptogenic signals arising from DNA damage to mitochondria, by causing cytochrome c release, presumably by interaction with BAK. Our group have suggested that mitochondria are common sites for phosphorylation of kinases and other proteins [5], [12]. Indeed, Poderoso et al. [5] have demonstrated that there was an interaction between mitochondrial StAR and ERK1/2, and as a result of this binding ERK1/2 phosphorylated StAR at Ser 232. Ser 2 in H2A, and Ser 2 and Ser 48 in H4 are embedded in an ERK phosphorylation motif [33] and both histones also display putative ERK docking domains in their amino acid sequences (Fig. S6) [32], [33]. Thus, it is plausible that ERK may phosphorylate these histones. An interesting possibility is that phosphorylated H2A and H4 serve as “sensors” of available ATP and of normal electron transfer chain function and coupling to oxidative phosphorylation, thereby constituting informative molecules for activation of the cell cycle under proliferative conditions (Fig. 8). ERK might also be phosphorylated in this region, and thus would dimerize and translocate to the nucleus, possibly together with the histones.

We found that ERK also interacts with VDAC (Fig. 3), a potentially significant finding inasmuch as VDAC1 features Ser 104 embedded in an ERK phosphorylation motif [31] and a potential ERK docking domain (Fig. S6) [32], [33]. Thus, ERK may activate VDAC by phosphorylation. Activation of VDAC by other MAPKs has been reported; p38 MAPK inhibition significantly reduced the phosphorylation of VDAC1 in a rabbit model of myocardial ischemia and reperfusion [41]. Further studies based on these findings are required to assess the implications for the regulation of cellular responses of ERK1 complexation with histones and VDAC in the mitochondrial compartment.

Mitochondrial transcription factor A (TFAM) stimulates mtDNA transcription *in vitro*, *in vivo* and *in organello* [42]–[44]. We observed a tendency for upregulation of almost every mitochondrial gene by both microarray and Real Time PCR. However, we failed to establish a statistical significance to these findings. TFAM contains in its amino acid sequence a potential ERK docking domain (Fig. S6) [32], [33] and Ser 31, Ser 177 and Thr 122 immersed in an ERK phosphorylation motif. ERK moves in and out mitochondria potentially through VDAC, and once in the mitochondrial matrix it could associate with TFAM to activate mtDNA transcription (Fig. 8).

We detected the formation of complexes of ERK1 with 2 enzymes of lipid metabolism: the fatty acid synthase (FAS) and the hydroxyacyl-Coenzyme A dehydrogenase (HAD). ERK1 also formed complexes with the β subunit of the mitochondrial ATP synthase F1 complex and peroxiredoxin 3 (Prx3, Table S2). Activation or inhibition of FAS and HAD may result in the modulation of AcCoA mitochondrial concentration, which is a potent regulator of mitochondrial oxidations. Further, we propose that ERK may have a modulatory role on antioxidant enzymes such as Prx3, as well as on ATPase, resulting in the regulation of oxidative phosphorylation and thereby of superoxide anion and mitochondrial H_2_O_2_ production (Fig. S8).

Galli *et al.* [12] showed that ERK1/2 exposure to low H_2_O_2_ concentration favoured the interaction with MEK, while higher doses impeded this binding capacity. They also observed that two cysteine residues in ERK2 structure were oxidized at the low H_2_O_2_ concentration. Here we show that oxidation of ERK1 impairs dimerization (Fig. 6D), whereas association with mitochondria favours the formation of the dimer (Fig. 6C). Philipova and Whitaker [18] argued that cofactors are required to achieve the formation of ERK dimers *in vitro*. We suggest that if cofactors are needed, they are best recruited in the proximity of mitochondria. Native ERK forms dimers and high ERK kinase activity is largely associated with biphosphodimers and not with monophosphodimers or phosphorylated monomers [18]. If we further consider that the OMM is enriched in ATP, and that non-posphorylatable ERK2 mutants escape from this region with slower kinetics [12], it follows that ERK phosphorylation and dimerization may occur in this compartment, and that this process may potentiate the translocation of the molecule to the nucleus (Fig. 8). It has been demonstrated that residues P176-D179 and L333, L336, L341 and L344 are essential for ERK2 homodimerization [13]. Most of these residues are conserved in ERK1. We and others [18] observed the formation of a covalent bond between ERK1 molecules. How this phenomenon correlates with the previous structural data remains to be elucidated.

From an evolutionary perspective, the structural and functional interactions of ERK with mitochondrial proteins constitute “modern” events. Bacteria, the ancestors of mitochondria, have a structurally and functionally diverse microbial kinome that includes histidine kinases, protein kinases-like enzymes and threonine kinases but not ERK1/2 or Akt [45]. Tyrosine kinases (TK) and TK receptors appeared later in unicellular eukariotes such as protists. The unicellular choanoflagellate *Monosiga brevicollis* has already an elaborate TK signaling network, even more complex than that of multicellular metazoans in which TK represent key evolutionary factors for intercellular signalling [46]. Further specialization of kinomes in complex organisms like fungi, nematodes or fly, arises in immunity, cell cycle modulation, differentiation, neurogenesis or intercellular communication [47]. Recent studies confirmed that MAPKs are more ancient than previously thought. ERK has been recognized in the protist *Dunaliella viridis* [48], in yeasts [47] and in the parasite *Entamoeba histolytica* [49]. ERK variants are present in insects, like *Drosophila*, in reptiles and mammals. In yeast, ERK presents a peptide in the C terminal tail, which is lost during evolution. This peptide leads to ERK being continuously phosphorylated and thus devoid of the regulation of modern ERK [50]. Modulation of ERK phosphorylation is likewise associated with evolution to multicellular organisms. In these and other publications [10], [12] we propose the existence of a mitochondrial ERK and phospho-ERK pool which depend on cytosol ERK expression and on cell stimulation by growth factors or stress, and argue that the phosphorylation and redistribution of this kinase also depends on trafficking to/from mitochondria. If so, the phenomenon would expand still further the spatio-temporal network regulating signal transduction effected by this key molecule [51].

In the present study we addressed the effects exerted by ERK1 on mitochondria of HeLa cells, as well as the potential roles of the organelle in determining ERK availability for the remaining cellular compartments. The results suggest novel, previously unrecognized functions of ERK and provide an integrated framework for defining and understanding novel ERK1 signalling pathways dependent on mitochondria in HeLa cells and perhaps more generally.

## Materials and Methods

### Cell culture

Human cervical carcinoma HeLa cells (obtained from Thomas Tuschl's laboratory, Department of Cellular Biochemistry, Max Plank Institute for Biophysical Chemistry, Göttingen, Germany), were cultured in D-MEM plus GlutaMAX™-1 (Gibco) with 10% FCS, streptomycin and penicillin, at 37°C in 5% CO_2_. The medium was replaced twice per week. Passages were made by trypsinization of confluent monolayers.

### Constructs and recombinant proteins

hERK1-GFP was a gift of Diane Lidke. Human ERK1-pcDNA3 cDNA was a gift of Dr. Jacques Pouyssegur. hERK1 was cloned into the EcoRI site of the bacterial expression vector pGEX-4T-1 and transformed into *E. coli* BL21. Recombinant GST-hERK1 protein was expressed and purified as described [52] with some modifications. Briefly, 100 ml of overnight culture was diluted 1:10 in a final volume of 1000 ml and incubated for 1 h at 37°C with shaking; 0.1 mM isopropyl 8-D-thiogalactoside was added, and the mixture was incubated further for 8 hr at 37°C with shaking. Bacteria were centrifuged at 6000**×** *g* for 5 min at 4°C. Pellets were lysed in 100 ml of 25 mM Tris-HCl, pH 7.7, 100 mM NaCl, 1 mM DTT, 0.1% NP-40 and 20% glycerol plus protease inhibitors (Protease inhibitor tablets, EDTA free, Roche). After 30 min on ice, cells were sonicated and centrifuged at 17,200**×** *g* for 15 min at 4°C. The supernatant was incubated with 1 ml of 50% v/v GSH-agarose beads (Sigma) overnight at 4°C with gentle rocking. The beads were washed four times with 10 volumes of PBS. The fusion protein was eluted with 3 volumes of 5 mM GSH, 50 mM Tris-HCl, pH 8.0. Free GSH was removed by dialysis against PBS. GST was removed from the recombinant protein with thrombin. The purified recombinant protein identity was corroborated by MALDI-TOF-MS and by western blot with specific antibodies and stored at −20°C.

hERK1-Dronpa was generated by cloning Dronpa (the kind gift of A. Miyawaki) [53] in frame with the hERK1 sequence in the hERK1-pcDNA3 vector.

### Mitochondrial isolation

HeLa cells were lysed in MSHE buffer (0.22 M mannitol, 0.07 M sucrose, 0.5 mM EGTA, 2 mM HEPES-KOH, pH 7.4) in the presence of protease inhibitors as above, and phosphatase inhibitors (25 mM NaF, 1 mM sodium orthovanadate). The homogenate was centrifuged for 10 min at 700**×** *g*. The supernatant was centrifuged at 7000**×** *g* for 20 min, and the mitochondrial pellet was washed once in 150 mM KCl, and once in MSHE. The mitochondrial fraction was essentially free of other compartmental contaminants (Fig. S1), as previously reported [54], [55]. Washing with 150 mM KCl depleted mitochondria of surrounding cytosolic mRNAs. For the microarray experiments, cytosolic mRNAs were needed for normalization and thus this washing step was omitted from the purification procedure.

### GST affinity pulldown assay

GST-based affinity pulldown assays were performed as previously reported [56], [57]. Briefly, 1 µg of GST-hERK1 or GST-null recombinant protein was immobilized on GSH-agarose beads, and incubated with 500 µg HeLa mitochondrial protein in binding buffer (0.2% Triton X-100, 150 mM NaCl, 20 mM Tris-HCl, pH 7.4, 1 mM EDTA, 1 mM EGTA, 0.2 mM sodium orthovanadate, and protease inhibitors as above), overnight at 4°C. GST-null protein was used as a negative control. The GST-hERK1 or GST-null protein complexes were extensively washed and resolved on SDS-PAGE. After colloidal Coomassie staining, either gel plugs of 1.5 mm diameter or entire gel bands were excised manually. A corresponding sample was excised from the negative control lane and processed in parallel. Only proteins not appearing in the negative control and found repeatedly in at least 2 pulldown experiments were considered as binding partners of hERK1. Five independent pulldown assays were performed.

### Protein identification

Manually excised gel plugs were subjected to an automated platform for the identification of gel-separated proteins [58] as described in the framework of recent large-scale proteome studies [59], [60]. An Ultraflex MALDI-TOF mass spectrometer (Bruker Daltonics) was used to acquire both peptide mass fingerprint (PMF) and fragment ion spectra, resulting in confident protein identifications based on peptide mass and sequence information. Database searches in the NCBI non-redundant primary sequence database restricted to the taxonomy *Homo sapiens* were performed using the Mascot Software 2.0 (Matrix Science) with parameter settings described earlier [59], [60]. The minimal requirement for accepting a protein as identified was at least one peptide sequence match above identity threshold in coincidence with at least four peptide masses assigned in the PMF.

Nano-flow LC-MS/MS was used as a complementary approach for protein identification, mainly to confirm the detection of histones. Tryptic peptides were subjected to reversed phase separation using 0.1% formic acid as solvent A and 0.1% formic acid in 100% acetonitrile as solvent B. An Ultimate nano-HPLC system (Dionex) consisting of an autosampler, a loading pump, and a nano-HPLC gradient pump connected to a 75-µm-inner diameter column (C_18_ PepMap, 15-cm length, 5-µm particle size, and 100-Å pore size) was utilized. Peptides were desalted and concentrated on a 300-µm-inner diameter C_18_ PepMap column of 1 mm length at a flow rate of 25 µl/min. Separation was performed with a flow rate of 250 nl/min using a binary gradient starting at 5% solvent B rising to 60% in 50 min. Peptides were directly eluted into an ESI ion trap LCQ Deca XP Plus mass spectrometer (Thermo) with the acquisition duty cycle set to a full scan mass spectrum (m/z 400–1500) followed by two data-dependent MS/MS scans. Mascot data base searches were performed as for the MALDI-TOF-MS data but the mass tolerance was 1.5 Da for the precursor ions and 1.0 Da for the fragment ions. The protein was considered identified when at least two peptide sequence matches above identity threshold were present.

### Disulfide bridge mapping

Gel bands were digested according to Shevchenko *et al.* [61] in the presence or absence of DTT. Dissolved samples were injected on an Ultimate gradient LC-System (LC Packings) equipped with pre-columns (25**×**0.075 mm packed in-house, C18, 5 µm, 300A (Vydac, Hesperia, USA)) working in back-flush mode. Peptides were separated with a standard gradient using 0.1% TFA in water as solvent A and 80% acetonitrile (v/v) in 0.1% TFA as solvent B, at a 300 nL/min flow rate on an analytical column (200**×**0.75 mm, C18, 5 µm, 300A (Vydac, Hesperia, USA)). The eluate was mixed with 10 mg/mL-cyano-4-hydroxycinnamic acid containing 10 fmol/µl Glu-Fibrinogen (Sigma) as internal standard in 70% acetonitrile, 0.1% TFA and spotted (Probot, LC Packings) every 15 sec onto an ABI stainless-steel target (Applied Biosystems). MALDI-ToF/ToF analysis was carried out in a 4800 Analyzer (Applied Biosystems/MDS Sciex) in positive mode. For MS experiments, 800 laser shots were accumulated, and for MS/MS max. 2000 laser shots were accumulated for each precursor. The acceleration voltage was 1 kV and collision energy was set to 10^-6^ torr. Proteins were identified as above. Two missed cleavages and oxidation and carbamylation as variable modifications were allowed for in the search. Mass deviation was 100 ppm for MS and 400 mmu for MS/MS.

For ESI-LC-MS/MS, extracted peptides were separated on an Agilent 1100 chromatography system equipped with a pre-column (25**×**0.75 mm, C18, 5 µm, 120 A, Maisch, Munich, Germany) in line with the analytical column (250**×**0.075 mm, C18, 5 µm, 120 A, Maisch, Munich, Germany). Eluting peptides were sprayed into an Orbitrap XL mass spectrometer working in CID mode under standard conditions.

### Mitochondrial protein import assay and *in organello* RNA synthesis

The mitochondrial import assay was performed according to Komiya and Mihara [62] with modifications. Briefly, 200 µg of mitochondrial proteins were incubated in 10 mM MOPS-NaOH, 2.5 mM phosphate-NaOH, pH 7.2, 250 mM sucrose, 5 mM MgCl_2_, 80 mM KCl, 2 mM ATP, 1% BSA, 1.42 mg/ml NADH plus 1 µg hERK1, at 37°C, for 1 h. Samples were then washed and run on SDS-PAGE. When mitochondrial gene expression was being assessed, ATP was substituted by 1 mM ADP, and NADH by 10 mM glutamate and 2.5 mM malate [36]. After treatment, mitochondria were washed and resuspended in Trizol (Invitrogen) for RNA isolation.

### Fluorescence labeling and confocal microscopy

Cells were grown on Lab-Tek Chambered Borosilicate Coverglass System (Nunc) for *in vivo* experiments, or on coverslides when cells were further fixed and immuno-stained, and transfected with hERK1-GFP or hERK1-Dronpa using Lipofectamine 2000 (Invitrogen) according to manufacturer's instructions. Cells were stained with specific mitochondrial markers, MitoTracker RED CMXRos or MitoTracker Deep Red (Invitrogen) (100 nM, 45 min at 37**°**C), fixed in 4% paraformaldehyde, permeabilized and labelled against phospho-ERK1/2, histones or VDAC in 10 mM Tris-HCl, pH 7.0, 1% BSA, 0.05% Triton X-100. Secondary antibodies were conjugated with Cy3 or Cy5. When appropriate, labelled coverslides were washed in 0.1% Triton X-100 in PBS for 1 h under stirring previous to montage.

Isolated mitochondria were labelled with MitoTracker Deep Red (Invitrogen) (100 nM, 45 min at 37**°**C), fixed in 4% paraformaldehyde, permeabilized and labelled against phospho-ERK1/2 and a secondary antibody conjugated to Cy3 as above.

Images of cells transfected with hERK-GFP and labeled for histones or VDAC plus Cy3 were acquired in a Zeiss LSM 510-meta confocal laser scanning microscope using a 63×1.2 NA water immersion objective. Excitation and emission filters were as follows: GFP, 488 nm excitation, BP 520±12 nm emission; Cy3, 532 nm excitation, LP 585 nm emission. FRET was performed by acceptor photobleaching [63]. The FRET pair was GFP and Cy3. Cy3 fluorescence was bleached up to 70% by 200 interactions of 532 nm laser at maximum power. No bleaching of GFP was detected under this condition. Negative controls for FRET were performed on cells transfected with hERK1-GFP without antibodies and Cy3 labelling. An additional control for the specificity of the interaction was performed with cells transfected with GFP and further labelled against histones or VDAC and the Cy3 secondary antibody as above. The FRET efficiency (FRET) was determined as:
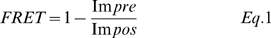
where Im*pre* and Im*pos* were GFP images before and after Cy3 photobleaching.

Other images were acquired with an Olympus FV-1000 confocal microscope using a 60×1.35 NA oil immersion objective. Excitation and filters were as follows: GFP, 488 nm excitation, BP 500–530 nm emission; MitoTracker RED CMXRos and Cy3, 543 nm excitation, BP 555–655 nm emission; MitoTracker Deep Red and Cy5, 633 nm excitation, BP 655–755 nm emission. Images were acquired in a sequential mode. No channel cross-talk was observed.

Endogenous phospho-ERK1/2 and histones or VDAC interaction was accomplished by FRET. In this case, we used Cy3/Cy5 as the fluorophore pair. Cy5 fluorescence was bleached up to 70% by 50 interactions of 633 nm laser at maximum power. No bleaching of Cy3 was detected in this condition. Negative controls were performed on cells labelled against phospho-ERK1/2 plus Cy3 and Cy5 for specificity, or only with Cy3 for a negative FRET value; or against VDAC plus Cy3 and Cy5 for specificity, and only with Cy3 for a negative FRET value. FRET was determined as Eq. 1; Im*pre* and Im*pos* were Cy3 images before and after Cy5 photobleaching.

### Image analysis

The image and statistical analyses were performed with Matlab (MathWorks, Natick, MA) and DIPimage (image processing toolbox for Matlab, Delft University of Technology, The Netherlands). Background levels were obtained by measuring the mean intensity of each signal outside the cells and were subtracted. Negative pixel values were clipped to zero.

To evaluate hERK1-GFP redistribution among the subcellular compartments of HeLa cells mitochondrial masks were generated from the MitoTracker fluorescence; nuclear masks were generated manually, and cellular masks of transfected cells were obtained using the GFP fluorescence. Further details of these procedures are supplied in the supporting online material (Fig. S9 and Supporting Information S1). hERK1-GFP fluorescence intensity was quantified in each compartment and normalized by the mean intensity of the whole cell or group of cells imaged simultaneously. Manders colocalization indexes, Manders Overlap Coefficient, and Pearson's correlation coefficient were calculated as previously described [64] (see also supporting online material).

The evaluation of phospho-ERK-Cy3 fluorescence intensity in isolated mitochondria was performed using a mask based on MitoTracker Deep Red fluorescence and the Cy3 signal within the mask.

FRET maps were generated using Eq. 1. The mean FRET efficiency within a mask was calculated using pixels with GFP (for the GFP/Cy3 FRET pairs) or Cy3 (for the Cy3/Cy5 FRET pairs) intensities exceeding the mean values of the entire image. Values were generated for every individual cell present in a given image and means were calculated from all the cells in all the images.

### Flow cytometry

Isolated mitochondria were fixed in 4% paraformaldehyde, permeabilized and labelled against histones in 10 mM Tris-HCl, pH 7.0, 1% BSA, 0.05% Triton X-100 or directly labelled without fixation and permeabilization in 10 mM Tris-HCl, pH 7.0, 1% BSA. Secondary antibody was conjugated with Cy5. Negative controls were performed by labelling only with secondary antibody. Mitochondria were analyzed on a Beckman Coulter Epics Elite flow cytometer.

### Western blot

Mitochondrial proteins and recombinant purified proteins were resolved on 10–12% SDS-PAGE gels (Invitrogen) and transferred onto nitrocellulose membranes. After blocking with 5% non-fat milk TTBS (0.1% Tween-20, 20 mM Tris-HCl, 137 mM NaCl, pH 7.4), the membranes were incubated with the proper primary (phospho-ERK from Cell Signaling, VDAC from Calbiochem, GAPDH from Santa Cruz, histones from Upstate and Complex I subunit from Molecular Probes) and secondary antibodies and detected with SuperSignal West Pico Chemiluminiscent Substrate (Pierce Biotechnology) with a Fujifilm LAS-1000 CCD camera.

### Microarray

Samples were DNAse I treated. RNA quality was determined using the Agilent 2100 Bioanalyzer (Agilent Technologies). The samples for hybridization were prepared from 0.75 µg of total RNA according to the Atlas SMART Fluorescent Probe Amplification Kit protocol (Clontech-Takara Bio Europe), except that (i) the RNA template was hydrolyzed under alkaline conditions before cDNA purification, and (ii) the PCR amplification process was monitored and terminated in the exponential phase. Quantity and dye incorporation rates of the converted RNA were assessed using a NanoDrop ND-100. Per microarray, 0.8 µg of Cy3 and Cy5 labelled cDNA fragments, respectively, were hybridized to Agilent Technologies 2**×**11K Custom Microarrays for 17 h at 63°C. The microarrays were designed via Agilent eArray and included 90 oligonucleotides for all known human mtRNAs (6 oligos for each gene). The rest of the DNA-chips were filled with selected genes from the human genome and were used for normalization. Post processing washes were performed according to the Agilent Technologies SSPE protocol (v2.1), replacing wash solution 3 by acetonitrile, followed by immediate scanning using an Agilent G2505B scanner. Intensity data were extracted with Agilent's Feature Extraction 9.5 software.

The oligos to map the mitochondrial genes were selected as follows: (i) more than one sequence was selected per gene; (ii) stretches of repeating oligonucleotides were avoided; (iii) a balanced AT and GC content was preferred; (iv) sense and antisense strands were considered; and (v) all sequences were blasted against GenBank database to check for cross hybridisation. Three oligos were selected (from the 5′-end, the middle and the 3′-end) of each mitochondrial gene sequence and translated into antisense. This procedure yielded six oligos per gene.

The raw intensity data were normalized with a non-linear lowess regression [65], [66]. Differentially expressed genes were identified by an ANOVA fixed effects model [67]. Adjusted *p*-values were obtained by the Benjamini-Hochberg method to control the false-discovery-rate [68].

The microarray data were generated conforming to the MIAME guidelines and deposited in the Gene Expression Omnibus (GEO) database at http://www.ncbi.nlm.nih.gov/geo/ (accession number GSE14963).

### Real Time PCR

cDNA was obtained as described above. Real Time PCR was performed with iQ SYBR Green supermix (Bio-Rad) in a iQ5 iCycler (BioRad) according to the manufacturer's instructions. Primers for each gene were either newly designed or taken from the Real Time PCR primer and probe Database (RTPrimerDB; http://medgen.urgent.be/rtprimerdb/) (Table S3). SemiQuantitative PCR was performed with One Step RT-PCR kit (Qiagen). Calibration curves were performed as described by manufacturer instructions. Efficiency accounted for more than 90%.

### Statistical analysis

Data are expressed as means±SE and analysed by one-way analysis of variance (ANOVA) and Scheffé test or the Student's *t* test. Statistical significance was accepted at *p*<0.05. hERK1-GFP redistribution upon FCS stimulation was evaluated in three image sequences. Redistribution with no stimulation was studied in one image sequence. hERK1-GFP localization to mitochondria in non-serum starved HeLa cells was determined in 6 images. Endogenous presence of ERK1/2 in mitochondria of HeLa cells was evaluated once by confocal microscopy and a minimum of twice by western blotting. The import assays based on western blotting was performed twice. The import assays for RNA isolation and semiquantitative PCR were performed at least three times and twice for Real Time. The FRET determination with hERK-GFP and histones or VDAC labelled with Cy3 was performed once; five images were taken and analysed for each interaction. The FRET measurements of endogenous phospho-ERK1/2 and histones or VDAC were repeated twice; >10 images were acquired and analysed for each interaction. The mean *FRET* values in each cell of a given image were determined and averaged. ERK dimer dissociation by β-ME was conducted three times with similar results. ERK dimer formation in the different subcellular compartments and the influence of oxidation were performed at least twice. The microarray determination was conducted once.

## Supporting Information

Supporting Information S1 This file contains supplementary results, methods, tables and references.
(0.05 MB DOC)

Figure S1 Identity and purity of mitochondrial fraction. Isolated mitochondria, cytosol, and nuclei were analysed to check for contamination. (A) Mitochondrial, nuclear and cytosolic fractions were labelled with MitoTracker Deep Red and analysed on a flow cytometer. Fluorescence intensity in arbitrary units. (B) Western blot of the same fractions with antibodies for characteristic proteins of mitochondria (Complex I) and cytosol (GAPDH). (C) DNA was extracted from mitochondria or whole cells and specific nuclear (GAPDH, Actin) and mitochondrial (ND1, ATP6) genes were detected by PCR. (D) RNA extracted from mitochondria (lower profile) or whole cell (upper profile) was DNAsed and analyzed on an Agilent 2100 Bioanalyzer microfluide electrophoresis. Peak profile of different size RNAs displayed on the right; corresponding generated band pattern of the RNA on the left. Nt = nucleotide, FU = Fluorescence Units.
(1.16 MB TIF)

Figure S2 hERK1 presence in mitochondria is dependent on cell condition. HeLa cells were transfected with hERK1-GFP, 24 h FCS starved or continuously grown in 10% FCS, and stained with MitoTracker CMXRos. Fluorescence intensity of both green (GFP) and red (MitoTracker) channels was analyzed in an Olympus FV1000 confocal microscope. Representative images of both channels separated and merged are shown. Bar = 10 µum. Yellow arrows indicate pixels that display MitoTracker fluorescence intensity but little or no GFP fluorescence intensity. Orange arrows indicate pixels that display both MitoTracker fluorescence and GFP fluorescence intensity.
(2.00 MB TIF)

Figure S3 Presence and translocation of hERK1 into mitochondria. (A) HeLa cells were transfected with hERK1-GFP, 24 h FCS starved and stained with MitoTracker CMXRos. Fluorescence intensity of both green (GFP) and red (Mitotracker) channels was followed for 20 min in an Olympus FV1000 confocal microscope without FCS stimulation of cells. Images of three representative time points of the individual and merged channels is shown. Bar = 10 µm. (B) Graph showing the redistribution of hERK1-GFP fluorescence intensity in the different cellular compartments in the absence of stimulus. (C) Change in hERK1-GFP fluorescence intensity in time analysed in mitochondria, nuclei and cytosol for each of the 3 confocal planes of the pair of images in A. Graphs show the net change displayed as percentage of the initial value (% of control) in each compartment in the absence of FCS stimulation. (D) Change in Pearson's correlation coefficient after FCS stimulation of serum starved cells analysed within the mitochondrial mask. (E) Zoom of the images in (A). Orange arrows indicate pixels that display both MitoTracker fluorescence and GFP fluorescence intensity.
(1.94 MB TIF)

Figure S4 Presence and translocation of GFP into mitochondria. (A) HeLa cells were transfected with GFP, serum starved for 24 h, and stained with MitoTracker Deep Red. Fluorescence intensity of both green (GFP) and red (Mitotracker) channels was followed for 10 min in an Olympus FV1000 confocal microscope upon 5% FCS stimulation of cells. Images of four representative time points of the individual and merged channels are shown. Bar = 10 µm. (B) Progress of hERK1-GFP fluorescence intensity in time analysed in mitochondria, nuclei and cytosol for each of the 3 confocal planes of the pair of images in A. Graphs show the net change displayed as the mean GFP fluorescence intensity of each compartment normalized by the mean GFP fluorescence of the cell.
(1.41 MB TIF)

Figure S5 Statistical analysis of hERK1 localization to mitochondria. Analysis was performed on the first pair of images of Fig. 1A. The probability distribution of random colocalization was obtained by computing the Pearson's correlation coefficient after repetitively scrambling the pixel positions in the green hERK1-GFP image. Red line = normal distribution adjusted to the data. The Pearson's correlation coefficient (P) of the original image is displayed in the inset and is far beyond the value in which the probability density curve equals 96% [21].
(0.53 MB TIF)

Figure S6 ERK docking sites. Potential ERK docking domains present in the ERK interactor partners. The N-terminal hydrophobic residue (green), the positively charged residues (blue), and the hydrophobic -X- hydrophobic motif (red) in the D motifs are indicated in accord to [32]. In bold, alternative consensus motif. Serine immersed in a phosphorylation consensus motif for ERK in TFAM sequence indicated in the box.
(0.49 MB TIF)

Figure S7 Histone recovery in the mitochondrial fraction. (A) Mitochondria of HeLa cells either fixed and permeabilized (medium panels), or without fixation and permeabilization (right panels) were labelled against histones and analysed by flow cytometry. Whole cells were fixed, permeabilized and labelled as a positive control (left panels). (B) Pure mitochondria were incubated with proteinase K, with or without Triton X-100 to permeabilize the organelle. Histones were recovered by acidic extraction and evaluated by western blot.
(0.71 MB TIF)

Figure S8 Proposed metabolic effects of ERK1 in mitochondria. In red, interaction confirmed by proteomics; in yellow, acetylated components; MTFA, mitochondrial transcription factor, FFA, free fatty acids.
(0.55 MB TIF)

Figure S9 Image analysis. (A) HeLa cells transfected with hERK1-GFP and stained with MitoTracker CMXRos. Bar = 10 µm. (B) Histogram of fluorescence intensity vs. number of pixels for both green (left) and red (right) channel images. Arrow indicates mean fluorescence intensity, also shown in the inset. (C) Mitochondrial compartment mask selected when MitoTracker fluorescence intensity was above twice the mean of the whole red image (upper right panel). The cellular compartment mask delimited when GFP fluorescence intensity was over the mean fluorescence intensity of the whole green image (upper left panel). Nuclear masks were determined manually (lower left panel). hERK1-GFP kinetics was studied only in mitochondria of transfected cells, and thus a new mask was determined by the combination of both the cellular mask and the Mitoctracker generated mask (lower right panel).
(0.78 MB TIF)

Table S1 Contamination indexes. 5′ nucleotidase and glucose-6-phosphatase were determined in mitochondria from HeLa cells and compared with existing data from the literature to asses for the contamination index of this fraction with endoplasmic reticulum. *A Percoll gradient was not used in the purification of mitochondria. ND: not determined. Data are mean±SD.
(0.03 MB DOC)

Table S2 hERK1 signaling complexes in mitochondria from HeLa cells. GST-hERK1 or GST-null recombinant proteins immobilized on GSH-agarose were incubated overnight with mitochondrial extract. Beads were extensively washed and eluted proteins run on PAGE-SDS. Proteins were stained with Coomasie Brilliant Blue G-250 (Fig. 2). A sample of each band in the GST-hERK1 or GST-null lane was excised and analysed by mass spectrometry for protein identification. Only proteins present in GST-hERK but not in GST-null precipitates were considered as specific ERK partners. These ERK interaction partners were indentified in at least 2 pull down experiments followed by MALDI-TOF-MS analysis.
(0.04 MB DOC)

Table S3 Real Time PCR primer pairs. ND: NADH dehydrogenase; ATP: ATP synthase; COX: cytochrome oxigenase; CYTB: cytochrome b. Number accounts for subunit. Primers were either newly designed (New) or extracted from the Real Time PCR primer Data Bank (RTPrimerDB)(http://medgen.urgent.be/rtprimerdb/).
(0.03 MB DOC)

Movie S1 Presence and translocation of hERK1 into mitochondria upon FCS stimulation. HeLa cells were transfected with GFP-hERK1, 24 h FCS starved and stained with MitoTracker CMXRos. Fluorescence intensity of both green (GFP) and red (Mitotracker) channels was followed for 20 min in an Olympus FV1000 confocal microscope after FCS stimulation of cells. Images were acquired every one minute.
(15.73 MB AVI)

Movie S2 Presence and translocation of hERK1 into mitochondria without stimulation. Same as in Movie S1 but without FCS stimulation of cells.
(7.87 MB AVI)
