# Supplementary material for: A New Paradigm for MAPK: Structural Interactions of hERK1 with Mitochondria in HeLa Cells
Source: PLoS One. 2009 Oct 22;4(10):e7541. doi: 10.1371/journal.pone.0007541 (PMC2760858; doi:10.1371/journal.pone.0007541)
Supplement: Supporting Information S1 — This file contains supplementary results, methods, tables and references. (0.05 MB DOC) [file pone.0007541.s001.doc]

**A new Paradigm for MAPK: Structural Interactions of**

**hERK1 with Mitochondria in HeLa Cells**

**Soledad Galli^1,2,*^, Olaf Jahn^3,4^, Reiner Hitt^5^, Doerte Hesse^3^,**

**Lennart Opitz^5^, Uwe Plessmann^6^**, **Henning Urlaub^6^, Juan Jose Poderoso^7^**

**Elizabeth A. Jares-Erijman^2^ and Thomas M. Jovin^1*^**

Submission of revised manuscript PLoS One 09-PONE-RA-08591-R1

**Supporting online material**

**Supporting information**

Integrity and purity of the mitochondrial fraction

The integrity and purity of the mitochondrial fraction were corroborated by different assays shown in Figure S1 and Table S1. The identity of the mitochondria was corroborated by transmission electron microscopy (TEM) and flow cytometry using MitoTracker Deep Red (Fig. S1A), and by western blot with antibodies directed against the 39 kDa subunit of mitochondrial complex I (Fig. S1B) and VDAC1 (not shown). The mitochondria showed no morphological signs of damage and were essentially free of other contaminants. No whole cells or nuclei were seen by TEM after the mitochondrial isolation protocol, and only a small membranous contamination attributable to microsomes was observed (data not shown). The whole mitochondrial population was positive for MitoTracker staining (Fig. S1A). The mitochondrial fraction was also essentially free of cytoplasmic contaminants such as GAPDH (absent by western blot, Fig. S1B), and the cytosolic enzyme lactate dehydrogenase. The activity of the latter enzyme accounted for < 20% of that found in cytosol (mitochondria 160±20* μM/min. mg protein; cytosol 950±270 μM/min. mg protein, **p*<0.05, Student’s *t* test, n=3). We obtained DNA from whole cells and from the mitochondrial fraction, and found no genomic genes in the DNA extracted from the mitochondria (Fig. S1C). We also extracted RNA from cells (total RNA) or from mitochondria (mtRNA). The electrophoretic pattern of total RNA was as expected; we observed the two peaks corresponding to the 28s and 18s RNA with mRNAs occupying intermediate positions (Fig. S1D). With respect to mtRNA we observed the following peculiarity: if mitochondria were not washed with KCl during the isolation protocol, the pattern of mtRNA resembled that of total RNA. However, after washing with 150 mM KCl we did not observe the 28s and 18s RNA lanes and mtRNA displayed a discrete number of peaks, probably representing the few genes encoded by mtDNA (Fig. S1D). To check for microsome contamination, we measured the activity of 5´nucleotidase and glucose-6-phosphatase, two enzymes typically used to assess for this type of cross contamination. A comparison of our values with those in the literature revealed significantly less activity in (our) HeLa mitochondria than in the mitochondria of rat liver, and much less than in microsomes from the different tissues (Table S1). Different authors have reported that the contamination of their fractions with microsomal enzymes was < 5% in all cases [71, 72], including purification protocols in which no Percoll gradient was used [73] (Table S1). Together, these results attest to the purity of our mitochondrial fractions, i.e., that they were substantially free of cytosolic, nuclear or microsomal components. The integrity and purity of similar mitochondrial preparations has also been reported elsewhere [10, 12, 70].

Colocalization coefficients

**Coefficients M and C evaluate the proportion of the pixels selected in one fluorescence channel that also display fluorescence in the other channel, and *vice versa* [64]. Thus, two M and C coefficients (M_green_, M_red_, C_green_ and C_red_) can be determined. Since we generated a mask according to the Mitotracker fluorescence intensity, all of the pixels inside the mask will display red fluorescence, and thus determined only the percentage of those pixels which also showed fluorescence intensity in the green channel (Fig. 1E, M and C). Pearson´s correlation coefficient and Manders Overlap coefficient were estimated as described [64].**

**The pixel size in these images was 137 nm × 137 nm. According to the optics, the minimal resolution gained with the microscope settings employed was 175 nm (lateral resolution = 0.4 × λ_em(cm)_/NA). Mitochondrial size ranges from 1-10 μm. Thus, even the widespread distribution of hERK1-GFP in the cell, the exhaustive analysis of the images argues for a true colocalization of the protein within the organelle. Together, the study of colocalization supports the idea that ERK specifically localizes to mitochondria of HeLa and its redistribution can be modulated.**

Energy metabolism enzymes

We detected the formation of complexes of ERK1 with 2 enzymes of lipid metabolism: the fatty acid synthase (FAS) and the hydroxyacyl-Coenzyme A dehydrogenase (HAD). FAS catalyses the synthesis of long chain fatty acids from acetyl-CoA, malonyl-CoA and NADPH [72], has a reported molecular mass of 273 kDa and is located in the cytosol [73]. Considering the fact that β-oxidation of fatty acids occurs inside mitochondria such that fatty acids must be imported into the organelle, the recovery of the complex ERK-FAS in a pure mitochondrial fraction suggests a close association of FAS with the OMM. The kinetics of reaction of FAS with acetyl coenzyme A (AcCoA) as well as FAS deacetylation have been well described [74]. The maximum number of acetyl groups bound to the enzyme has been estimated in 3.8 mol/mol. These groups are rapidly removed from the native enzyme by hydroxylamine. Spontaneous hydrolysis of FAS occurs at 23 ºC with a rate constant of 4.7×10^-4^ s^-1^. Thus, FAS is central to set the mitochondrial content of its substrate AcCoA [74].

HAD is a member of the short-chain dehydrogenase/reductase superfamily and catalyzes the oxidation of a wide variety of fatty acids, alcohols, and steroids in mitochondrial fatty acid β-oxidation [75]. HAD, contrarily to FAS, promotes degradation of fatty acids and increases AcCoA level. Both FAS and HAD have putative sites for phosphorylation by ERK1; FAS has 17 serines and 11 threonines and HAD has one threonine. All of these residues are in minimal phosphorylation consensus motifs. It is noteworthy that ERK1 may form complexes with one enzyme of lipid biosynthesis and another enzyme of lipid biodegradation, thereby exerting opposing effects on these proteins with a variable outcome depending on local factors. Considering that AcCoA is a potent regulator of mitochondrial oxidations, activation or inhibition of FAS and HAD may result in the modulation of AcCoA mitochondrial concentration. Thus, ERK might be indirectly involved in the regulation of mitochondrial oxidations by modulation of the mitochondrial pool of AcCoA (Fig. S7).

ERK1 also formed complexes with the β subunit of the mitochondrial ATP synthase F1 complex and peroxiredoxin 3 (Prx3). The former enzyme has a primary role in ATP synthesis, while the Prx3 belongs to a family of multifunctional antioxidant thioredoxin-dependent peroxidases, the major functions of which include cellular protection against oxidative stress, modulation of intracellular signalling cascades that utilize hydrogen peroxide as a second messenger molecule, and regulation of cell proliferation [76]. ATPase β subunit has no putative Ser or Thr target for phosphorylation by ERK1 and thus further evidence is needed to establish an influence of the kinase on this protein. There is extensive evidence for important effects of ROS on the activation of MAPKs. Low levels of H_2_O_2_ lead to ERK activation, whereas higher levels activate apoptotic signalling modules [12, 54, 55]. In this context we propose that ERK may have a modulatory role on antioxidant enzymes such as Prx3, as well as on ATPase, resulting in the regulation of oxidative phosphorylation and thereby of superoxide anion and mitochondrial H_2_O_2_ production (Fig. S7).

Structural proteins

We also repeatedly detected the association of ERK1 with tubulin in mitochondria. Microtubules are involved in many cellular processes, including the transport of proteins and organelles, the establishment of cell polarity and the formation of the meiotic and mitotic spindles. Through the formation of these complexes, ERK could enable mitochondrial transport mediated by the cytoskeleton.

**Supplementary Methods**

Image analysis

We performed a histogram of fluorescence intensity vs. number of pixels for both green and red channel images and calculated the mean fluorescence intensity for each of them (Fig. S8B). Most of the pixels were background and displayed low fluorescence intensity. Thus, the mean fluorescence intensity of the whole image corresponded mainly to the intensity of background. To define the mitochondrial compartment we selected a mask determined by a MitoTracker fluorescence intensity twice above the mean of the whole image (Fig. S8C, upper right panel). The cellular compartment was delimited when GFP fluorescence intensity was over the mean fluorescence intensity of the whole image (Fig. S8C, upper left panel). Nuclear masks were performed manually (Fig. S8C, lower left panel). hERK1-GFP kinetics were studied only in mitochondria of transfected cells, and thus a new mask was determined by the combination of both the cellular mask and the MitoTracker generated mask (Fig. S8C, lower right panel). Cytosolic compartment was determined by the remaining cell area after subtracting mitochondrial and nuclear areas to the whole cell area. GFP fluorescence intensity change was evaluated in every compartment in time. For imaging in time, cells were stimulated with FCS and imaged every 1-2 min over a period of 20 min at room temperature; 3-8 equidistant (0.5 μm) planes were evaluated for each cell or group of cells (an image set corresponds to the images over time of all the planes, in both the green and red channels, for a cell or group of cells simultaneously imaged).

Intensity correlation analysis was studied as previously described [77].

Enzyme activities

For the detection of 5´nucleotidase activity, mitochondria were incubated in 100 mM Tris-HCl, pH 7.6, 10 mM MgCl_2_ and 5 mM AMP at 37 ºC for 5 min. For glucose-6-phosphatase activity determination, mitochondria were incubated in 100 mM Na-acetate buffer, pH 6.5, 20 mM glucose-6-phosphate and 10 mg/mL BSA, for 15 min at 37 ºC. Inorganic phosphate released from AMP and glucose-6-phosphate was measured as described by Itaya and Ui [78]. Lactate dehydrogenase activity was measured by following the change in NADH absorbance at 340 nm in 100 mM potassium phosphate buffer, pH 7, 1% Triton X-100, 1 mM sodium pyruvate, 200 μM NADH at 25 ºC.

Supplementary References

69. Bhagwat SV, Boyd MV, Ravindranath V (2000) Multiple forms of cytochrome P450 and associated monooxygenase activities in human brain mitochondria. Biochem Pharmacol 59: 573-582.

70. Converso DP, Taille C, Carreras MC, Jaitovich A, Poderoso JJ, Boczkowski J (2006) HO-1 is located in liver mitochondria and modulates mitochondrial heme content and metabolism. FASEB J 20: E482-E492.

71. Brunner G, Bygrave FL (1969) Microsomal marker enzymes and their limitations in distinguishing the outer membrane of rat liver mitochondria from microsomes. Eur J Biochem 8: 530-534.

72. [Wakil SJ](http://www.ncbi.nlm.nih.gov/entrez/query.fcgi?db=pubmed&cmd=Retrieve&dopt=AbstractPlus&list_uids=2669958&query_hl=49&itool=pubmed_docsum) (1989) Fatty acid synthase, a proficient multifunctional enzyme. Biochemistry 28: 4523-30.

73. [Jayakumar A, Tai MH, Huang WY, al-Feel W, Hsu M, et al.](http://www.ncbi.nlm.nih.gov/entrez/query.fcgi?db=pubmed&cmd=Retrieve&dopt=AbstractPlus&list_uids=7567999&query_hl=50&itool=pubmed_docsum) (1995) Human fatty acid synthase: properties and molecular cloning. Proc Natl Acad Sci USA 92: 8695-8699.

74. Cognet JA, Hammes GG (1983) Elementary steps in the reaction mechanism of chicken liver fatty acid synthase: acetylation-deacetylation. Biochemistry 22: 3002-3007.

75. [Yang SY, He XY, Schulz H](http://www.ncbi.nlm.nih.gov/entrez/query.fcgi?db=pubmed&cmd=Retrieve&dopt=AbstractPlus&list_uids=16176262&query_hl=51&itool=pubmed_docsum) (2005) 3-Hydroxyacyl-CoA dehydrogenase and short chain 3-hydroxyacyl-CoA dehydrogenase in human health and disease. FEBS J 272: 4874-4883.

76. [Immenschuh S, Baumgart-Vogt E](http://www.ncbi.nlm.nih.gov/entrez/query.fcgi?db=pubmed&cmd=Retrieve&dopt=AbstractPlus&list_uids=15890023&query_hl=26&itool=pubmed_docsum) (2005) Peroxiredoxins, oxidative stress, and cell proliferation. Antioxid Redox Signal 7: 768-77.

77. Qi L, Lau A, Morris T, Guo L, Fordyce C, Stanley E (2004) A syntaxin 1 Gα_0_ and N-type calcium channel complex at a presynaptic nerve terminal: analysis by quantitative immunocolocalization. J Neurosci 24: 4070-4081.

78. Itaya K, Ui M (1966) A new micromethod for the colorimetric determination of inorganic phosphate. Clin Chim Acta 14: 361-366.

79. Crain RC, Zilversmit DB (1981) Lipid dependence of glucose-6-phosphate phosphorylase: a study with purified phospholipid transfer proteins and phosphatidylinositol-specific phospholipase C. Biochemistry 20: 5320-5326.

80.Katewa SD, Katyare SS (2003) A simplified method for inorganic phosphate determination and its application for phosphate analysis in enzyme assays. Anal Biochem 323: 180-187.

81. Fujii J, Ikeda Y (2002) Advances in our understanding of peroxiredoxin, a multifunctional, mammalian redox protein. Redox Rep 7: 123-30.
