# Supplementary figures and images for: A New Paradigm for MAPK: Structural Interactions of hERK1 with Mitochondria in HeLa Cells
Source: PLoS One. 2009 Oct 22;4(10):e7541. doi: 10.1371/journal.pone.0007541 (PMC2760858; doi:10.1371/journal.pone.0007541)

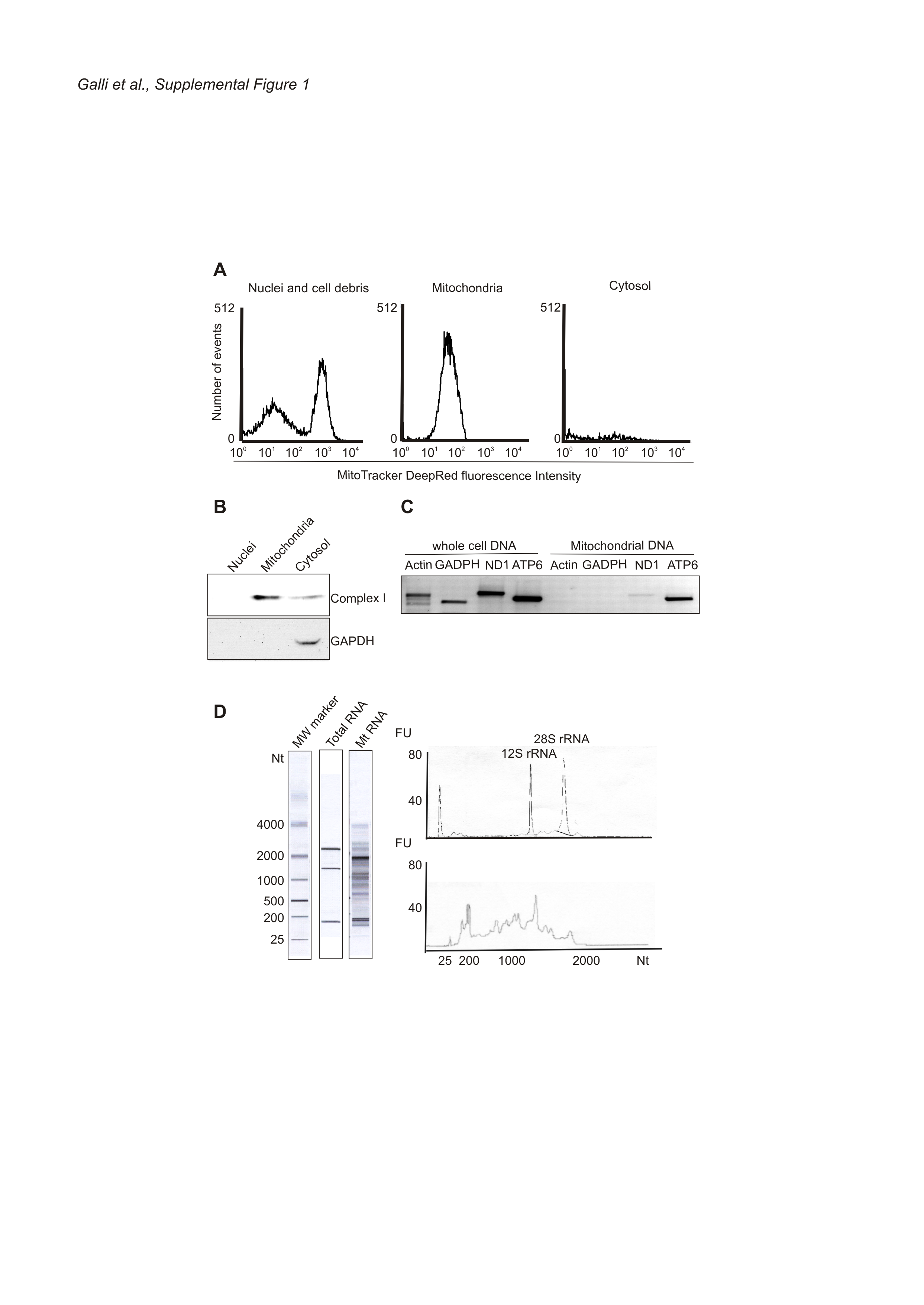

Supplement: Figure S1 — Identity and purity of mitochondrial fraction. Isolated mitochondria, cytosol, and nuclei were analysed to check for contamination. (A) Mitochondrial, nuclear and cytosolic fractions were labelled with MitoTracker Deep Red and analysed on a flow cytometer. Fluorescence intensity in arbitrary units. (B) Western blot of the same fractions with antibodies for characteristic proteins of mitochondria (Complex I) and cytosol (GAPDH). (C) DNA was extracted from mitochondria or whole cells and specific nuclear (GAPDH, Actin) and mitochondrial (ND1, ATP6) genes were detected by PCR. (D) RNA extracted from mitochondria (lower profile) or whole cell (upper profile) was DNAsed and analyzed on an Agilent 2100 Bioanalyzer microfluide electrophoresis. Peak profile of different size RNAs displayed on the right; corresponding generated band pattern of the RNA on the left. Nt = nucleotide, FU = Fluorescence Units. (1.16 MB TIF) [file pone.0007541.s002.tif]

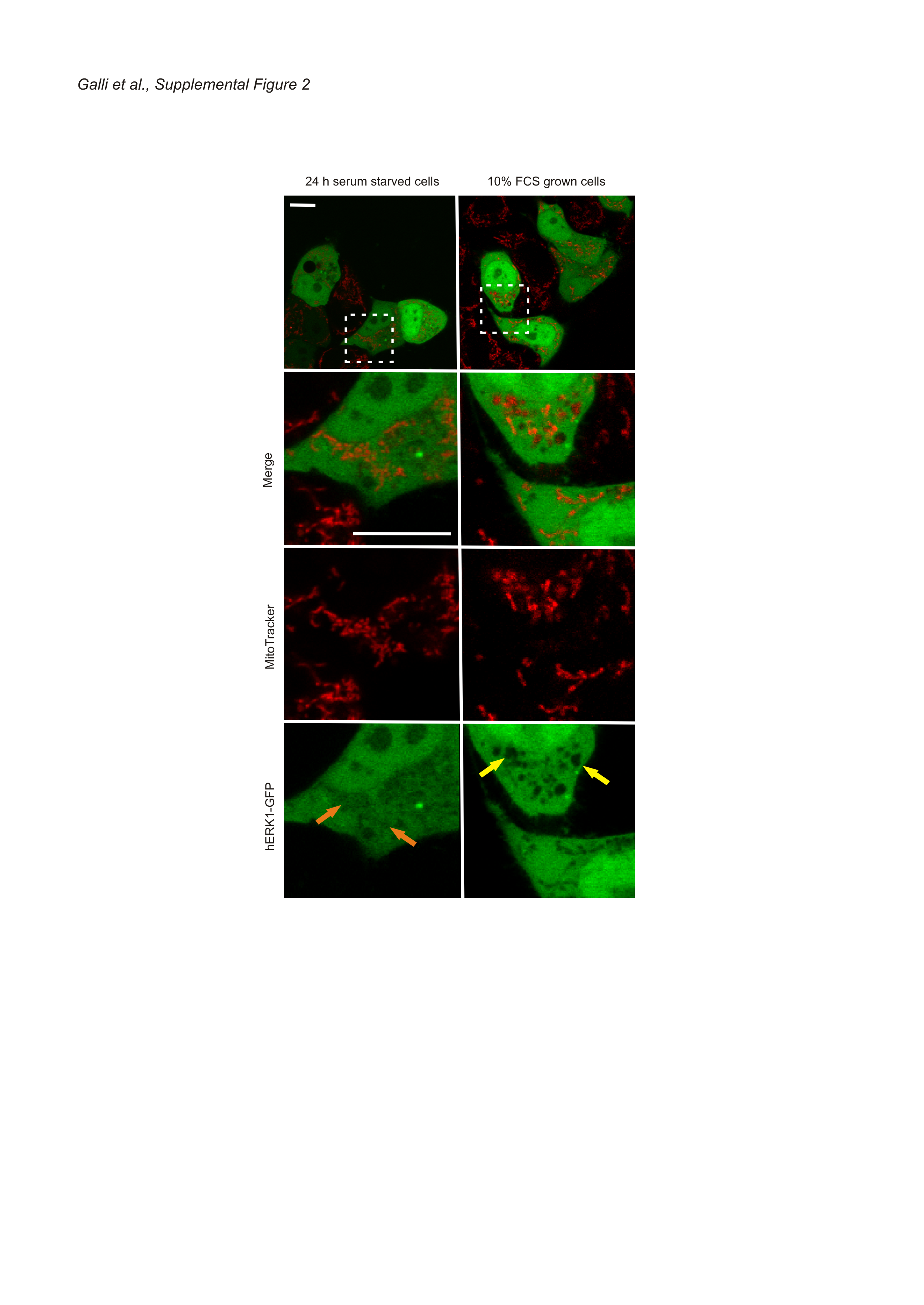

Supplement: Figure S2 — hERK1 presence in mitochondria is dependent on cell condition. HeLa cells were transfected with hERK1-GFP, 24 h FCS starved or continuously grown in 10% FCS, and stained with MitoTracker CMXRos. Fluorescence intensity of both green (GFP) and red (MitoTracker) channels was analyzed in an Olympus FV1000 confocal microscope. Representative images of both channels separated and merged are shown. Bar = 10 µum. Yellow arrows indicate pixels that display MitoTracker fluorescence intensity but little or no GFP fluorescence intensity. Orange arrows indicate pixels that display both MitoTracker fluorescence and GFP fluorescence intensity. (2.00 MB TIF) [file pone.0007541.s003.tif]

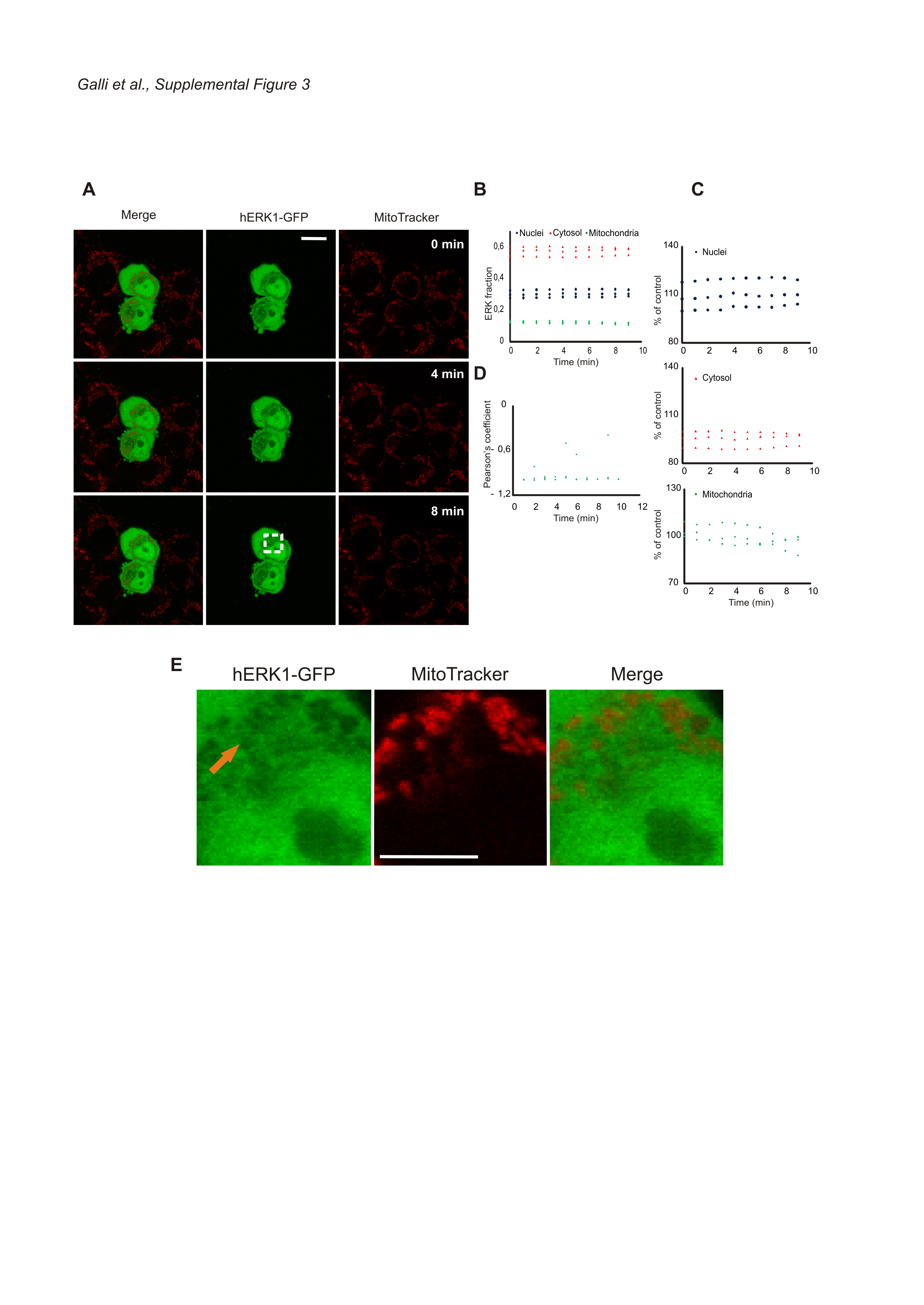

Supplement: Figure S3 — Presence and translocation of hERK1 into mitochondria. (A) HeLa cells were transfected with hERK1-GFP, 24 h FCS starved and stained with MitoTracker CMXRos. Fluorescence intensity of both green (GFP) and red (Mitotracker) channels was followed for 20 min in an Olympus FV1000 confocal microscope without FCS stimulation of cells. Images of three representative time points of the individual and merged channels is shown. Bar = 10 µm. (B) Graph showing the redistribution of hERK1-GFP fluorescence intensity in the different cellular compartments in the absence of stimulus. (C) Change in hERK1-GFP fluorescence intensity in time analysed in mitochondria, nuclei and cytosol for each of the 3 confocal planes of the pair of images in A. Graphs show the net change displayed as percentage of the initial value (% of control) in each compartment in the absence of FCS stimulation. (D) Change in Pearson's correlation coefficient after FCS stimulation of serum starved cells analysed within the mitochondrial mask. (E) Zoom of the images in (A). Orange arrows indicate pixels that display both MitoTracker fluorescence and GFP fluorescence intensity. (1.94 MB TIF) [file pone.0007541.s004.tif]

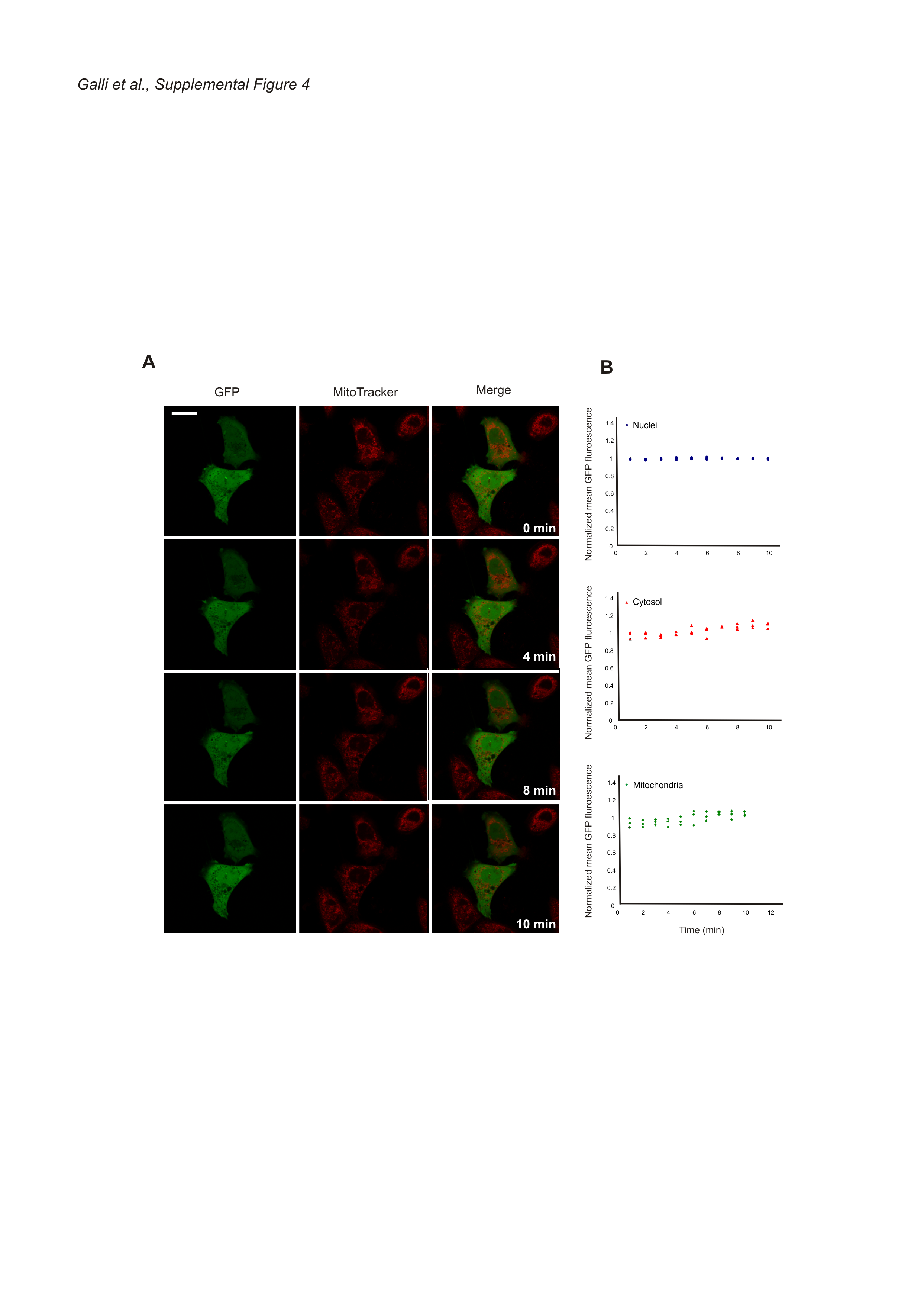

Supplement: Figure S4 — Presence and translocation of GFP into mitochondria. (A) HeLa cells were transfected with GFP, serum starved for 24 h, and stained with MitoTracker Deep Red. Fluorescence intensity of both green (GFP) and red (Mitotracker) channels was followed for 10 min in an Olympus FV1000 confocal microscope upon 5% FCS stimulation of cells. Images of four representative time points of the individual and merged channels are shown. Bar = 10 µm. (B) Progress of hERK1-GFP fluorescence intensity in time analysed in mitochondria, nuclei and cytosol for each of the 3 confocal planes of the pair of images in A. Graphs show the net change displayed as the mean GFP fluorescence intensity of each compartment normalized by the mean GFP fluorescence of the cell. (1.41 MB TIF) [file pone.0007541.s005.tif]

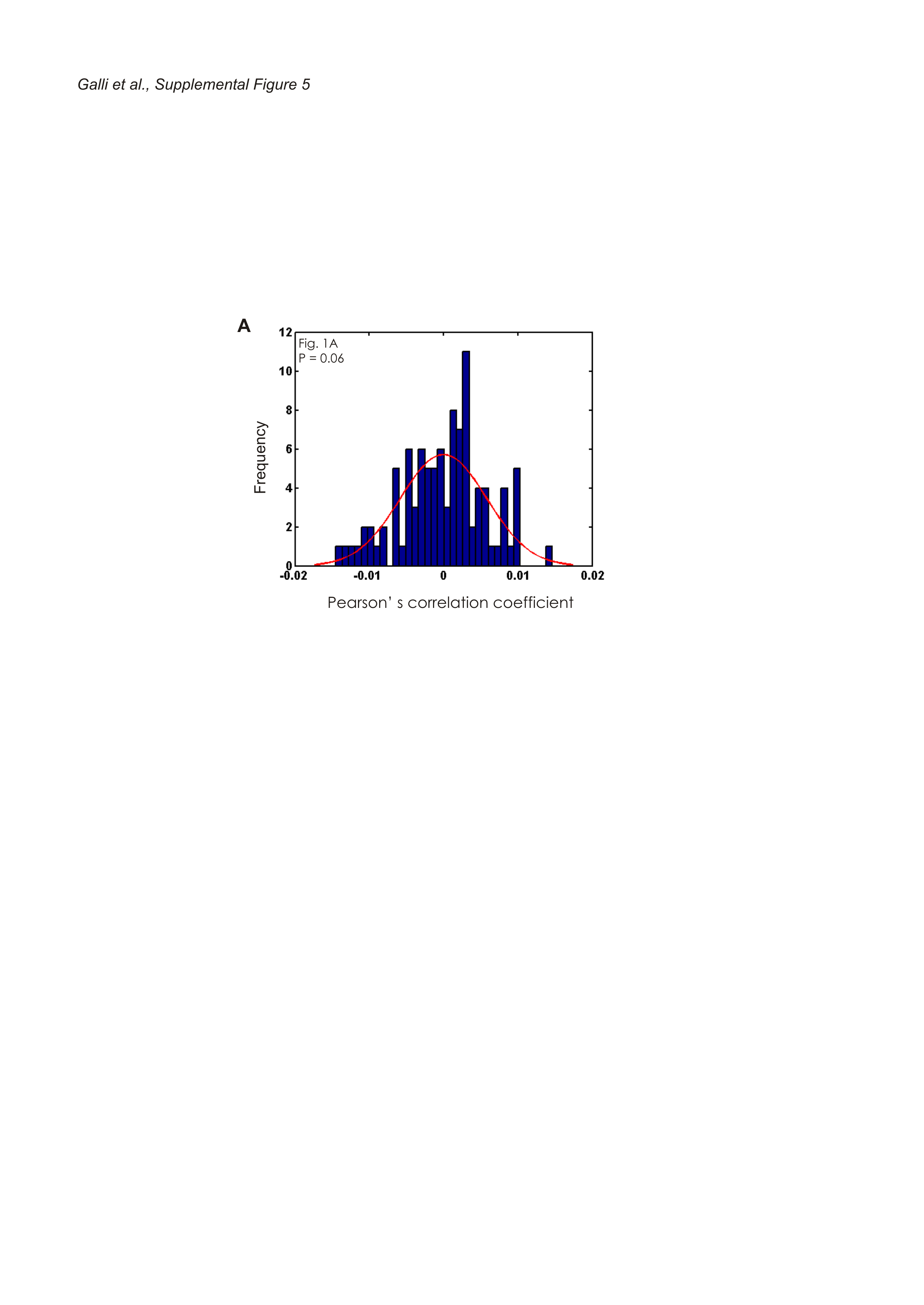

Supplement: Figure S5 — Statistical analysis of hERK1 localization to mitochondria. Analysis was performed on the first pair of images of Fig. 1A. The probability distribution of random colocalization was obtained by computing the Pearson's correlation coefficient after repetitively scrambling the pixel positions in the green hERK1-GFP image. Red line = normal distribution adjusted to the data. The Pearson's correlation coefficient (P) of the original image is displayed in the inset and is far beyond the value in which the probability density curve equals 96% [21]. (0.53 MB TIF) [file pone.0007541.s006.tif]

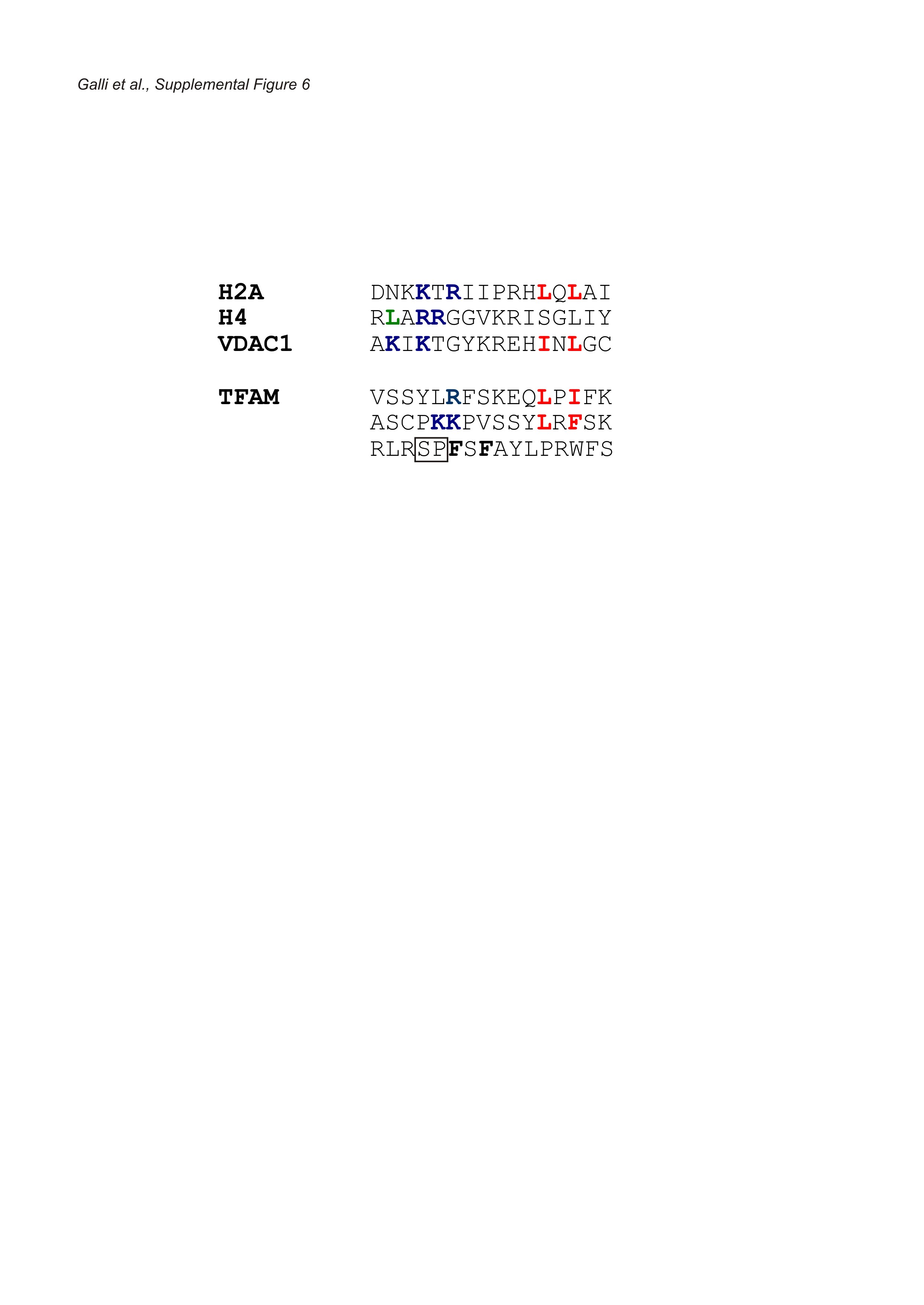

Supplement: Figure S6 — ERK docking sites. Potential ERK docking domains present in the ERK interactor partners. The N-terminal hydrophobic residue (green), the positively charged residues (blue), and the hydrophobic -X- hydrophobic motif (red) in the D motifs are indicated in accord to [32]. In bold, alternative consensus motif. Serine immersed in a phosphorylation consensus motif for ERK in TFAM sequence indicated in the box. (0.49 MB TIF) [file pone.0007541.s007.tif]

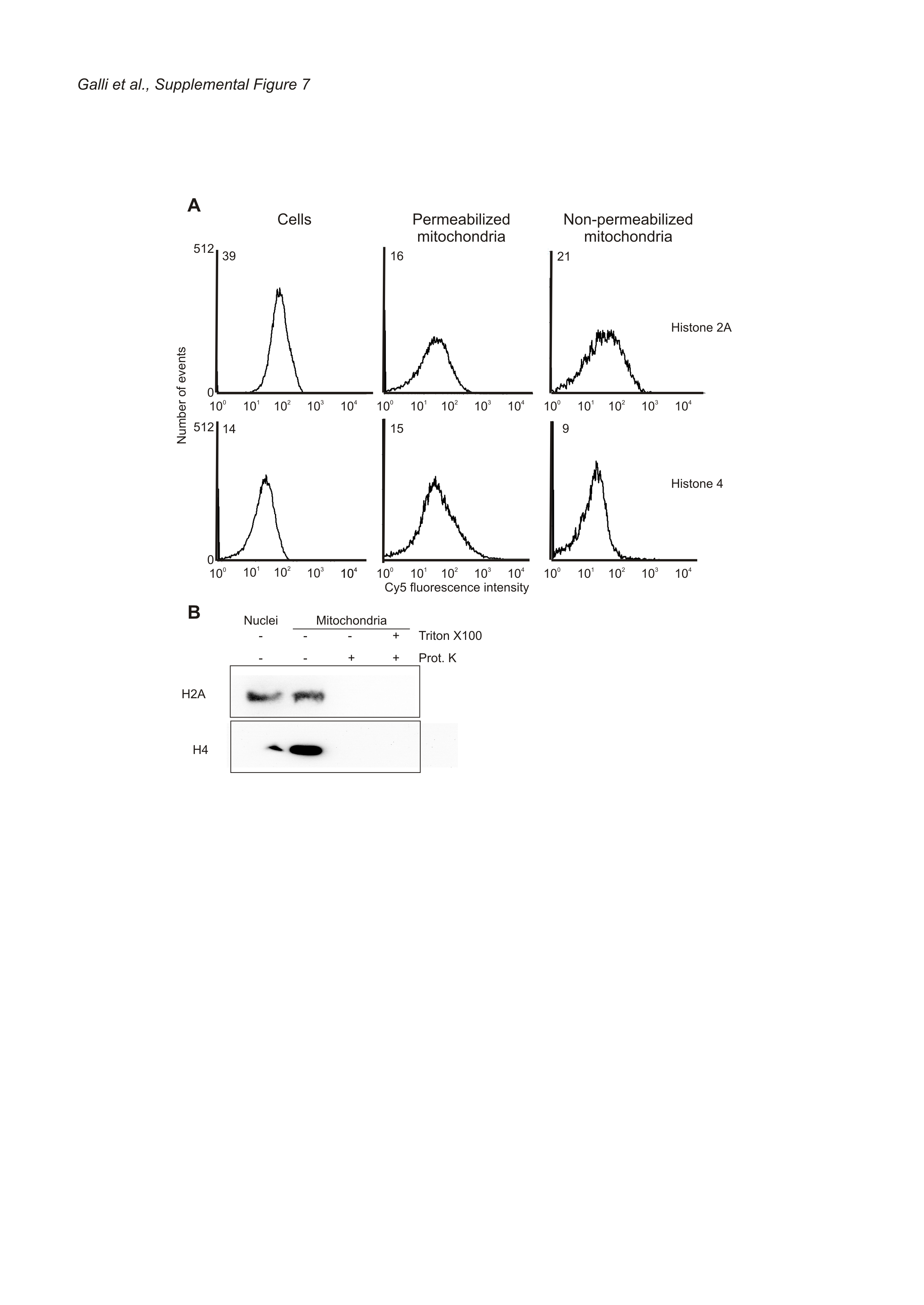

Supplement: Figure S7 — Histone recovery in the mitochondrial fraction. (A) Mitochondria of HeLa cells either fixed and permeabilized (medium panels), or without fixation and permeabilization (right panels) were labelled against histones and analysed by flow cytometry. Whole cells were fixed, permeabilized and labelled as a positive control (left panels). (B) Pure mitochondria were incubated with proteinase K, with or without Triton X-100 to permeabilize the organelle. Histones were recovered by acidic extraction and evaluated by western blot. (0.71 MB TIF) [file pone.0007541.s008.tif]

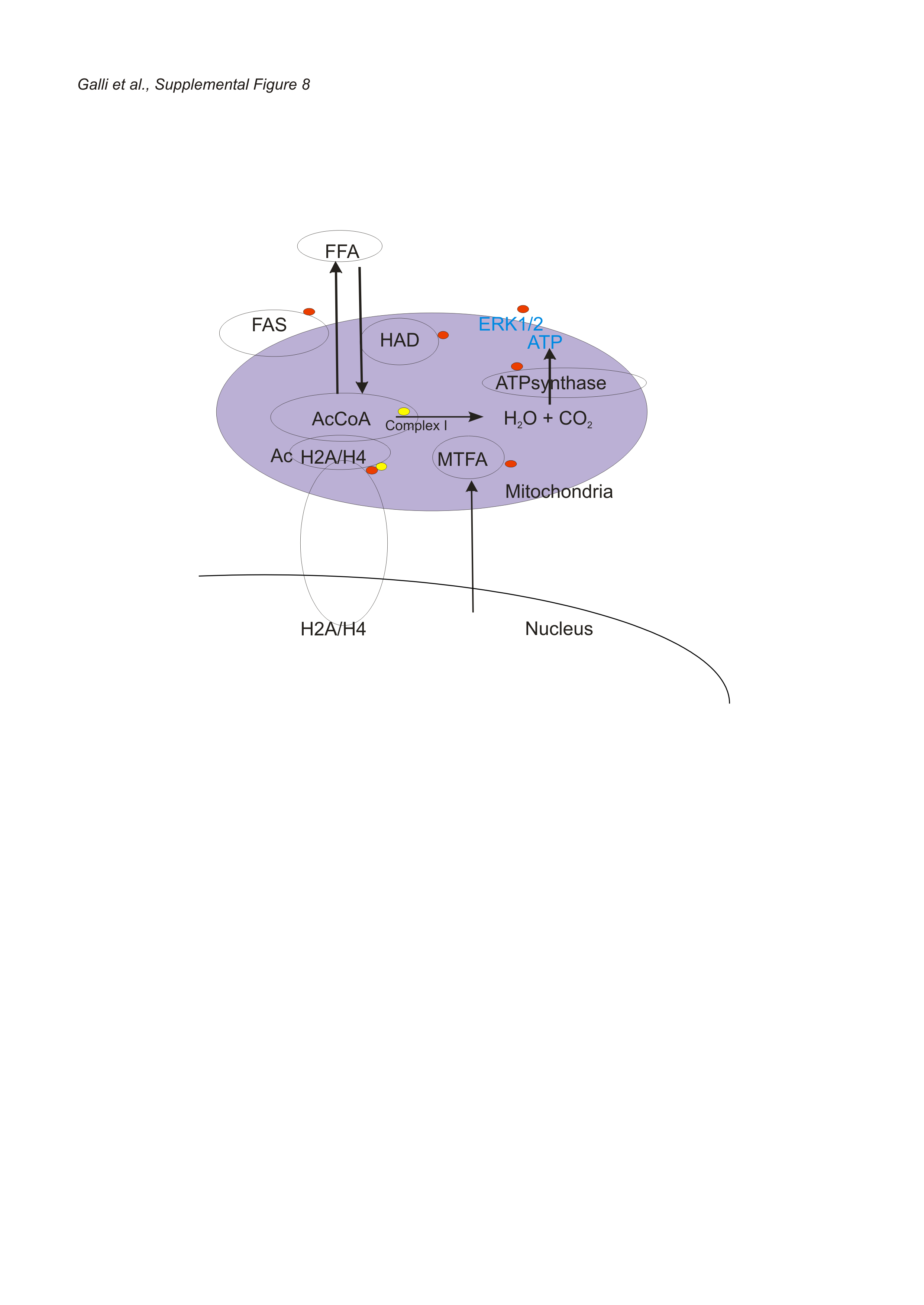

Supplement: Figure S8 — Proposed metabolic effects of ERK1 in mitochondria. In red, interaction confirmed by proteomics; in yellow, acetylated components; MTFA, mitochondrial transcription factor, FFA, free fatty acids. (0.55 MB TIF) [file pone.0007541.s009.tif]

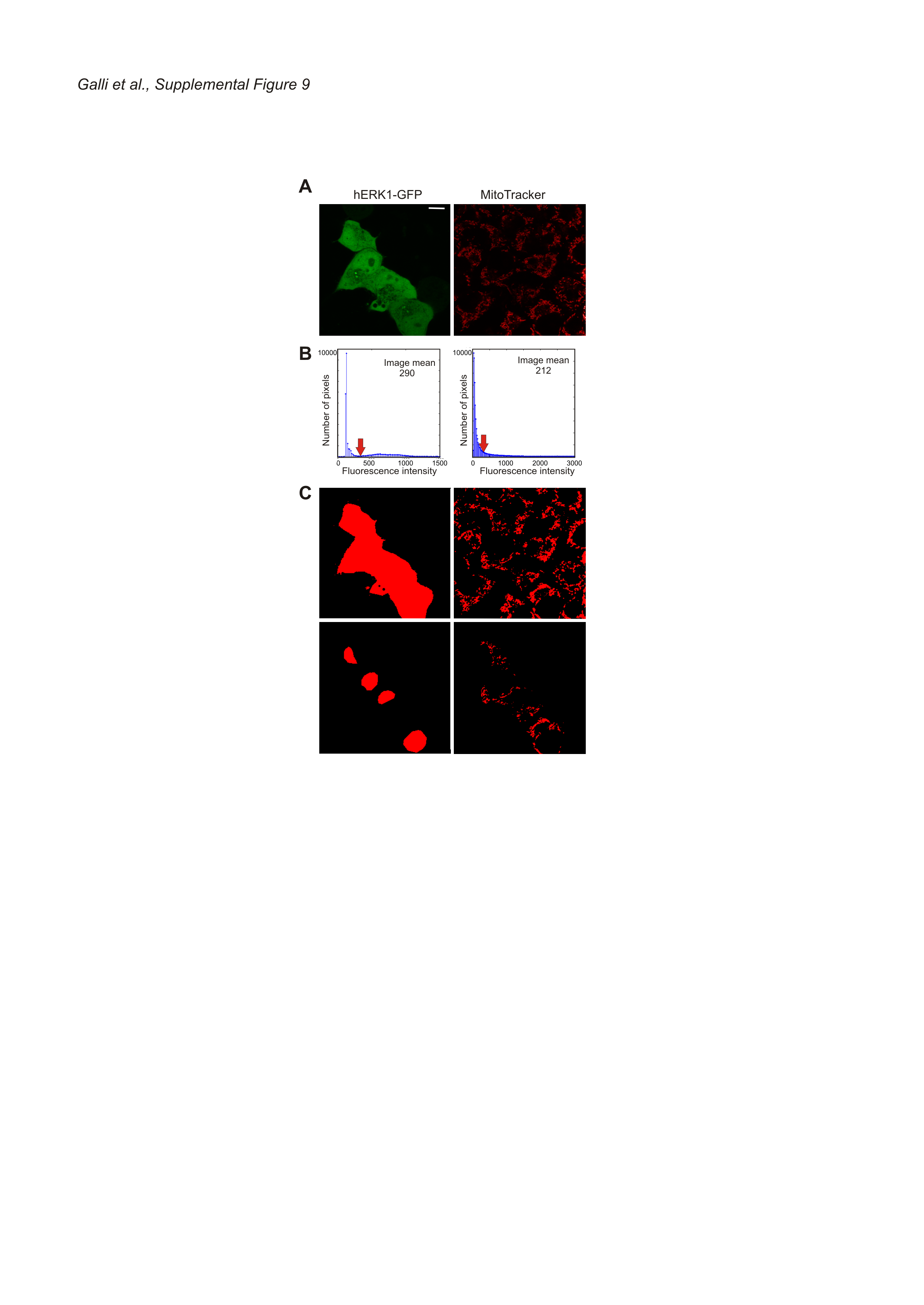

Supplement: Figure S9 — Image analysis. (A) HeLa cells transfected with hERK1-GFP and stained with MitoTracker CMXRos. Bar = 10 µm. (B) Histogram of fluorescence intensity vs. number of pixels for both green (left) and red (right) channel images. Arrow indicates mean fluorescence intensity, also shown in the inset. (C) Mitochondrial compartment mask selected when MitoTracker fluorescence intensity was above twice the mean of the whole red image (upper right panel). The cellular compartment mask delimited when GFP fluorescence intensity was over the mean fluorescence intensity of the whole green image (upper left panel). Nuclear masks were determined manually (lower left panel). hERK1-GFP kinetics was studied only in mitochondria of transfected cells, and thus a new mask was determined by the combination of both the cellular mask and the Mitoctracker generated mask (lower right panel). (0.78 MB TIF) [file pone.0007541.s010.tif]
