# Supplementary material for: A New Paradigm for MAPK: Structural Interactions of hERK1 with Mitochondria in HeLa Cells
Source: PLoS One. 2009 Oct 22;4(10):e7541. doi: 10.1371/journal.pone.0007541 (PMC2760858; doi:10.1371/journal.pone.0007541)
Supplement: Table S1 — Contamination indexes. 5′ nucleotidase and glucose-6-phosphatase were determined in mitochondria from HeLa cells and compared with existing data from the literature to asses for the contamination index of this fraction with endoplasmic reticulum. *A Percoll gradient was not used in the purification of mitochondria. ND: not determined. Data are mean±SD. (0.03 MB DOC) [file pone.0007541.s011.doc]

**Table S1:** Contamination indexes

| 5´nucleotidase (μmol/min.mg protein) | Mitochondria | Microsome | % contamination | Reference |
| --- | --- | --- | --- | --- |
| HeLa  Rat Liver | 0.00025±0.0001 0.1± 0.09 | ND 10±1 | ND 1 | [70] |
| Glucose-6-phosphatase (nmol/ min.mg protein) |  |  |  |  |
| HeLa  Rat Liver  Human Brain | 0.62±0.11  27  5.2 | ND  710 411 84±8  448 | ND  3.8*  1.2 | [71] [79] [80]  [69] |

5´nucleotidase and glucose-6-phosphatase were determined in mitochondria from HeLa cells and compared with existing data from the literature to asses for the contamination index of this fraction with endoplasmic reticulum. *A Percoll gradient was not used in the purification of mitochondria. ND: not determined. Data are mean± SD.
