# Supplementary material for: A New Paradigm for MAPK: Structural Interactions of hERK1 with Mitochondria in HeLa Cells
Source: PLoS One. 2009 Oct 22;4(10):e7541. doi: 10.1371/journal.pone.0007541 (PMC2760858; doi:10.1371/journal.pone.0007541)
Supplement: Table S2 — hERK1 signaling complexes in mitochondria from HeLa cells. GST-hERK1 or GST-null recombinant proteins immobilized on GSH-agarose were incubated overnight with mitochondrial extract. Beads were extensively washed and eluted proteins run on PAGE-SDS. Proteins were stained with Coomasie Brilliant Blue G-250 (Fig. 2). A sample of each band in the GST-hERK1 or GST-null lane was excised and analysed by mass spectrometry for protein identification. Only proteins present in GST-hERK but not in GST-null precipitates were considered as specific ERK partners. These ERK interaction partners were indentified in at least 2 pull down experiments followed by MALDI-TOF-MS analysis. (0.04 MB DOC) [file pone.0007541.s012.doc]

***Table S2:*** *hERK1 signaling complexes in mitochondria from HeLa cells.*

|  | **MW (kDa)** | **Known function** | **Accesion No** | **Cellular Localization** |
| --- | --- | --- | --- | --- |
| Metabolic enzymes |  |  |  |  |
| FAS, fatty acid synthase | 275.5 | Synthesis of long chain fatty acids from acetyl- CoA, malonyl- CoA and NADPH. | [gi|915392](http://mascot.em.mpg.de/mascot/cgi/protein_view.pl?file=E:/Proteomics/sgalli/20061024147484.dat&hit=gi|915392&px=1&protscore=45.82&_mudpit=1000) | Cytosol [73] |
| Hydroxyacyl-Coenzyme A dehydrogenase, type II isoform 2 (HAD) | 26.2 | Catalyzes the oxidation of a wide variety of fatty acids, alcohols, and steroids in mitochondrial fatty acid β-oxidation. | [gi|83715985](http://mascot.em.mpg.de/mascot/cgi/protein_view.pl?file=E:/Proteomics/sgalli/20061024147366.dat&hit=1) | Mitochondria |
| Peroxiredoxin 3 (Prx3) isoform precursor | 28 | Cellular protection against oxidative stress, modulation of intracellular signalling cascades that apply hydrogen peroxide as a second messenger molecule, and regulation of cell proliferation [81]. | [gi|14250063](http://mascot.em.mpg.de/mascot/cgi/protein_view.pl?file=E:/Proteomics/sgalli/20061024147475.dat&hit=gi|14250063&px=1&protscore=128.67&_mudpit=1000) | Mitochondria |
| Mitochondrial ATP synthase, H+ transporting F1 complex beta subunit | 48 | ATP synthesis. | [gi|89574029](http://mascot.em.mpg.de/mascot/cgi/protein_view.pl?file=E:/Proteomics/sgalli/20061024147329.dat&hit=6) | Mitochondria, inner mitochondrial membrane. |
| Protein kinases |  |  |  |  |
| ERK1 | 44 | Signalling pathways generally involved with cell proliferation and differentiation. | gi|31221 | Mitochondria, cytosol, nuclei |
| Structural proteins |  |  |  |  |
| Tubulin | 50 | Cytoskeleton | [gi|223429](http://mascot.em.mpg.de/mascot/cgi/protein_view.pl?file=E:/Proteomics/sgalli/20061024147473.dat&hit=gi|223429&px=1&protscore=43.56&_mudpit=1000) | Cytoskeleton |

GST-hERK1 or GST-null recombinant proteins immobilized on GSH-agarose were incubated overnight with mitochondrial extract. Beads were extensively washed and eluted proteins run on PAGE-SDS. Proteins were stained with Coomasie Brilliant Blue G-250 (Fig. 2). A sample of each band in the GST-hERK1 or GST-null lane was excised and analysed by mass spectrometry for protein identification. Only proteins present in GST-hERK but not in GST-null precipitates were considered as specific ERK partners. These ERK interaction partners were indentified in at least 2 pull down experiments followed by MALDI-TOF-MS analysis.
