# Supplementary material for: A New Paradigm for MAPK: Structural Interactions of hERK1 with Mitochondria in HeLa Cells
Source: PLoS One. 2009 Oct 22;4(10):e7541. doi: 10.1371/journal.pone.0007541 (PMC2760858; doi:10.1371/journal.pone.0007541)
Supplement: Table S3 — Real Time PCR primer pairs. ND: NADH dehydrogenase; ATP: ATP synthase; COX: cytochrome oxigenase; CYTB: cytochrome b. Number accounts for subunit. Primers were either newly designed (New) or extracted from the Real Time PCR primer Data Bank (RTPrimerDB)(http://medgen.urgent.be/rtprimerdb/). (0.03 MB DOC) [file pone.0007541.s013.doc]

**Table S3: Real Time PCR primer pairs.**

| **Mitochondrial gene** | **Primer pair** | **Source** |
| --- | --- | --- |
| ND1  ND2  ND3  ND4  ND4L  ND5  ND6  ATP6  ATP8  COX1  COX2  COX3  CYTB  12S RNA  16S RNA | Forward: 5´ ATACCCCCGATTCCGCTACGAC 3´  Reverse: 5´ GTTTGAGGGGGAATGCTGGAGA 3´  Forward: 5´ attccatccaccctcctctc 3´  Reverse: 5´ tggggtgggttttgtatgtt 3´  Forward: 5´ ccctccttttacccctacca 3´  Reverse: 5´ ggccagacttagggctagga 3´  Forward: 5´ ctcgctaacctcgccttacc 3´  Reverse: 5´ agtgagccccattgtgttgt 3´  Forward: 5´ taaccctcaacacccactcc 3´  Reverse: 5´ ggccatatgtgttggagattg 3´  Forward: 5´ CAAAACCTGCCCCTACTCCT 3´  Reverse: 5´ GGGTTGAGGTGATGATGGAG 3´  Forward: 5´ gggtggtggttgtggtaaac 3´  Reverse: 5´ ccccgagcaatctcaattac 3´  Forward: 5´ CGCCACCCTAGCAATATCAA 3´  Reverse: 5´ TTAAGGCGACAGCGATTTCT 3´  Forward: 5´ atggcccaccataattaccc 3´  Reverse: 5´ gcaatgaatgaagcgaacag 3´  Forward: 5´ CGATGCATACACCACATGAA 3´  Reverse: 5´ AGCGAAGGCTTCTCAAATCA 3´  Forward: 5´ tgaagcccccattcgtataa 3´  Reverse: 5´ acgggccctatttcaaagat 3´  Forward: 5´ ggcatctacggctcaacatt 3´  Reverse: 5´ cgaagccaaagtgatgtttg 3´  Forward: 5´ AATTCTCCGATCCGTCCCTA 3´  Reverse: 5´ GGAGGATGGGGATTATTGCT 3´  Forward: 5´ ggtcgaaggtggatttagca 3´  Reverse: 5´ ccatgttacgacttgtctcctc 3´  Forward: 5´ gggataacagcgcaatccta 3´  Reverse: 5´ cctggattactccggtctga 3´ | RTPrimerDB  New  New  New  New  RTPrimerDB  New  RTPrimerDB  New  RTPrimerDB  New  New  RTPrimerDB  New  New |

ND: NADH dehydrogenase; ATP: ATP synthase; COX: cytochrome oxigenase; CYTB: cytochrome *b*. Number accounts for subunit. Primers were either newly designed (New) or extracted from the Real Time PCR primer Data Bank (RTPrimerDB)(<http://medgen.urgent.be/rtprimerdb/>).
